# One-step strategy for fabricating icariin-encapsulated biomimetic Scaffold: Orchestrating immune, angiogenic, and osteogenic cascade for enhanced bone regeneration

**DOI:** 10.1016/j.bioactmat.2025.06.001

**Published:** 2025-06-10

**Authors:** Fengxin Zhao, Fuying Chen, Tao Song, Luoqiang Tian, Hang Guo, Dongxiao Li, Jirong Yang, Kai Zhang, Yumei Xiao, Xingdong Zhang

**Affiliations:** a National Engineering Research Center for Biomaterials, College of Biomedical Engineering, Research Center for Material Genome Engineering, Sichuan University, Chengdu, 610065, China; b Sichuan Academy of Chinese Medicine Science, Chengdu, Sichuan, 610042, China; c Research Center for Human Tissue and Organs Degeneration, Institute of Biomedical and Biotechnology, Shenzhen Institute of Advanced Technology, Chinese Academy of Sciences, Shenzhen, 518055, China

**Keywords:** Biomimetic scaffold, Bone regeneration, Icariin delivery, One-step strategy, Cascade activation

## Abstract

The repair of bone defects relies on the intricate coordination of inflammation, angiogenesis, and osteogenesis. However, scaffolds capable of integrating osteo-immunomodulation and vascular-bone coupling to cascade-activate these processes remain a challenge. Here, a biomimetic scaffold (CHP@IC) with *in situ* PLGA@icariin (PLGA@IC) microspheres encapsulation was successfully fabricated using a one-step emulsification and polymerization strategy. This approach not only simplifies the fabrication process but also ensures high encapsulation efficiency and sustained release of IC through PLGA@IC microspheres. The findings from subcutaneous implantation, network pharmacology-predicted molecular targets, and *in vitro* studies collectively reveal that the CHP@IC-induced M2 polarization of macrophages via STAT3 signaling pathway triggers the sequential activation of inflammation, angiogenesis, and osteogenesis to enhance bone regeneration. The CHP@IC scaffold exhibited a significant osteogenic advantage in cranial defect repair, yielding new bone volumes approximately 3-fold and 10-fold greater than those in the CHP group and blank control group, respectively. This study not only elucidates the mechanism of IC in promoting regeneration of bone but also provides a novel method for designing scaffolds aimed at the efficient repair of bone defects.

## Highlights

• A biomimetic scaffold (CHP@IC) with *in situ* PLGA@icariin microspheres encapsulation was fabricated using a one-step emulsification and polymerization strategy.
• This strategy not only simplifies the fabrication process but also ensures high encapsulation efficiency and sustained release of IC.
• CHP@IC-induced M2 polarization of macrophages via STAT3 signaling pathway triggers the sequential activation of inflammation, angiogenesis, and osteogenesis to enhance bone regeneration.

## Graphical abstract

**Figure:**
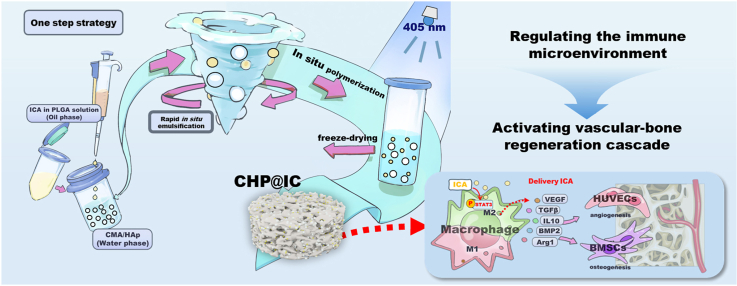

## 1 Introduction

Bone defects represent a significant clinical orthopedic problem, characterized by a high prevalence and being associated with a variety of underlying pathological or physiological factors [1]. They pose a serious threat to patients' lives and also increase the economic burden [2]. Although bone retains an inherent regenerative capacity, the self-healing process may fail once the bone defect exceeds a critical size [3]. In such cases, intervened treatment with the aid of a bone implant becomes necessary. The development of biomaterials provides patients with treatment options for the functional reconstruction of bone [[4], [5], [6]]. However, it was difficult to meet the numerous physiological demands of the bone regeneration process by relying on solely monofunctional biomaterials [7].

The process of bone repair is a meticulously orchestrated biological sequential event involving hemostasis, inflammation, stem cell homing, angiogenesis and osteogenesis [4]. A moderate inflammatory response holds great significance in the bone repair process, as it expedites the recruitment of cells at the defect site and promotes the secretion of cytokines associated with bone repair [8]. The discipline of osteoimmunology has demonstrated the close relationship between the immune system and the skeletal system, and it has been shown that the timely improvement of the immune microenvironment plays a critical role in biomaterial-mediated bone regeneration [9,10]. Moreover, the processes of angiogenesis and osteogenesis are two interrelated and crucial aspects of the bone regeneration process. The growth of blood vessels facilitates the supply of essential oxygen and nutrients to the defect site, as well as the rapid transportation of cytokines, which regulates the bone regeneration process [11,12]. Therefore, developing a scaffold integrates the abilities of osteo-immunomodulation and vascular-bone regeneration cascade is urgently needed [[13], [14], [15]].

The scaffolds motivated by exogenous factors or bioactive agents to regulate the biological processes of bone repair represent a viable clinical option [7,16]. Icariin (ICA) is a widely used osteogenic activity drug [17]. The current study reveals two main aspects of ICA in bone regeneration: a) Promotion of osteogenic differentiation, b) Inhibition of osteoclastogenesis. ICA promotes osteogenic differentiation of BMSCs through modulating the ERα-Wnt/β-catenin signaling pathway, PI3K-AKT signaling pathway and MAPK signaling pathway to up-regulate the expression of osteogenesis-related proteins (ALP, BMP2, RUNX2, OCN et al.) [[18], [19], [20]]. It also inhibits osteoclastogenesis by decreasing the expression of RANKL and NF-κB [21,22]. Furthermore, some studies have indicated that the potential of ICA to promote bone regeneration is related to its immunomodulatory ability: ICA alleviates osteoporosis by activating anti-inflammatory effects through autophagy [23]. And ICA also improves osteointegration and reduces osteolysis by reducing the immuno-inflammatory response induced by PEEK and titanium implants. [17,24]. However, the specific mechanism of this association and the process of bone repair events activated by ICA-mediated immune response are not clear. In addition, the physiological function and compatibility of ICA are concentration dependent [25]. Local concentrations higher than those required for immunomodulation and osteogenic differentiation (10^−8^-10^−5^ M) result in uncontrolled cytotoxicity [17,24,26]. However, bone repair is a process with a long therapeutic cycle, which necessitates an increase in drug content. Thus, increasing the loading of ICA in scaffolds and maintaining a safe and effective released concentration are challenging. Although both polymer scaffolds and 3D-printed scaffolds have been used for the delivery of ICA, the current methods still have difficulty simultaneously meeting the needs of sustained release and high drug loading within the bone repair treatment cycle [27,28]. Various microspheres with adjustable particle sizes and flexible application forms have been widely used as drug carriers because they can increase drug loading and balance the relationship between action concentration and therapeutic cycle [29,30]. Yuan et al. used MgO/MgCO_3_ particles to adsorb ICA and then encapsulated them in PLGA microspheres by emulsification, which could increase drug loading and reduce the early burst release of ICA [31]. However, the preparation and loading of microspheres into scaffolds in traditional methods are two separate processes. The microsphere preparation requires a time-consuming solvent evaporation step that results in considerable drug loss and increases the complexity of the overall preparation process [29].

**Scheme 1:**
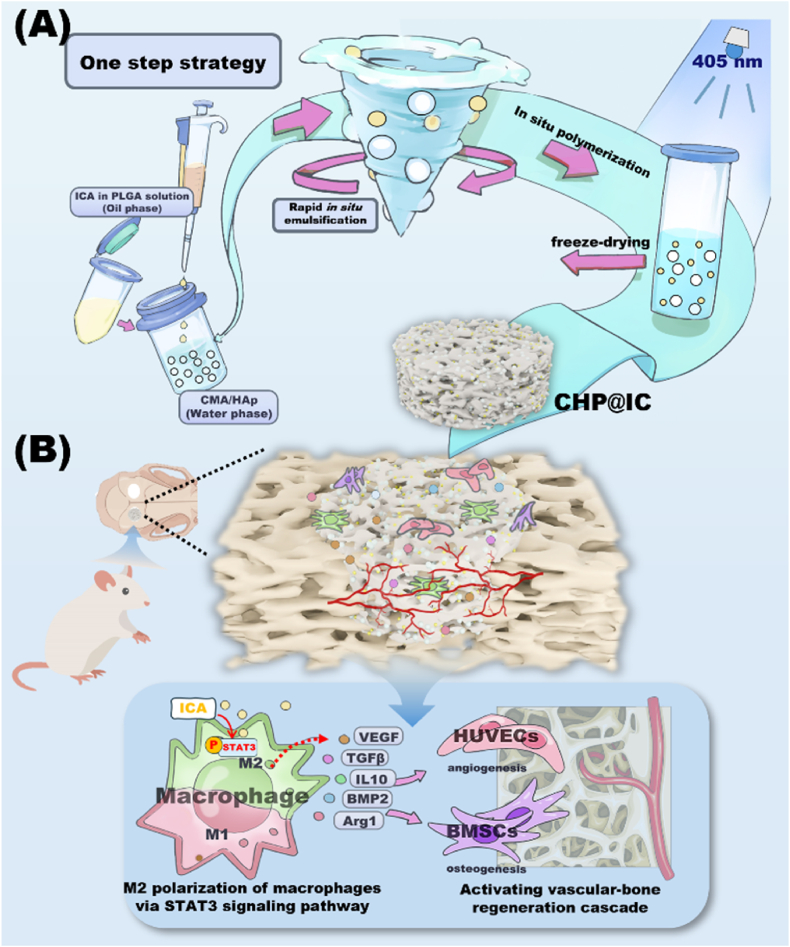
(A) One-step strategy prepared CHP@IC scaffold containing PLGA@IC microspheres. (B) CHP@IC activates immune-mediated vascular-bone regeneration cascade via STAT3 signaling pathway.

Biomimetic scaffolds consisting of collagen/hydroxyapatite, which can mimic the natural composition and/or structure of the natural bone, have become the most widely researched hotspot [32,33]. In this study, the methacrylate anhydride-modified collagen (CMA) and hydroxyapatite (HAp) were chosen as raw materials to simulate the natural bone organic/inorganic composition, providing an optimized bone microenvironment for bone repair [34]. In addition, thiolated icariin (ICA-SH, IC) synthesized in our previous study was also used, IC was able to reduce the sudden release and prolong the release cycle by utilizing intermolecular disulfide bonds as well as improve drug compatibility [21,22,35]. Here, a one-step strategy of *in situ* emulsification and polymerization was developed to construct an IC motivated CMA/HAp biomimetic scaffold (CHP@IC), which allows for *in situ* encapsulation of PLGA@IC microspheres to effectively improve encapsulation efficiency and sustained-release behavior of IC. To more fully understand the mechanism of action of CHP@IC in bone regeneration, the potential targets of ICA by network pharmacology were analyzed. Furthermore, the results *in vivo* and *in vitro* suggest that CHP@IC scaffold regulated the immune microenvironment and activated the vascular-bone regeneration cascade through the STAT3 signaling pathway (Scheme 1). This finding not only provides theoretical support for the application of ICA but also offers a novel method to design scaffolds for effective treatment of bone defects.

## 2 Materials and methods

### 2.1 Construction of CHP scaffold based on *in situ* emulsification and polymerization

To prepare methacrylate anhydride-modified collagen (CMA)/hydroxyapatite (HAp)/poly (lactic-co-glycolic acid) (PLGA) scaffold (CHP scaffold), CMA (372–388 kDa) was firstly synthesized according to our previous reports [36,37]. Then, it was dissolved in 0.2 M acetic acid, and the pH of CMA solution was adjusted to 7.4 by 1 M NaOH at 4 °C. Next, the HAp (25–40 μm) sintered at 800 °C for 2 h was added into the neutral collagen solution and mixed evenly. The mixed solution was considered as the water-phase. And PLGA (DG-50DLGH055, 53–70 kDa, Daigangbio, China) was dissolved in the dichloromethane (DCM, 99.8 %), which was termed as oil-phase. The oil-phase was added drop by drop to the water-phase at a volume ratio of 1:6.5, and the mixture was placed on a vortex oscillator to oscillate violently for 40 s to form the primary emulsion. Then the primary emulsion added 0.06 % (w/v) LAP photo-initiator (99.8 %, Jiangyin Stem Easy Biotech, Ltd, China) was immediately placed in blue light (405 nm) for 90 s to obtain hydrogels containing PLGA microspheres. Finally, the hydrogels were lyophilized to obtain the CHP scaffold (φ = 10 mm, h = 1.5 mm) after being cleaned in turn by 20 % acetone solution and deionized water. Meanwhile, CMA scaffold and CMA/HAp scaffold were also prepared by respectively polymerizing and lyophilizing collagen solution and CMA/HAp mixed solution. The final concentrations of CMA, HAp, and PLGA were 10 mg/mL, 100 mg/mL and 6 mg/mL, respectively.

CMA, CMA/HAp and CHP scaffolds were frozen in liquid nitrogen and subsequently their cross sections were coated with an ultrathin layer of gold via ion sputter deposition. The internal network structure and PLGA microspheres in scaffolds were observed by field emission scanning electron microscopy (SEM, Hitachi, S-4800). Furthermore, the CHP scaffolds underwent degradation in Tris-HCl solution containing collagenase I (C8140, Solarbio, China) at 37 °C and the precipitates were collected and washed. Subsequently, the precipitates were observed using a polarizing microscope (Zeiss, Germany) and the particle size of PLGA microspheres was statistically analyzed using Image J software (NIH, USA).

### 2.2 Construction and characterization of CHP@IC scaffolds

CMA/HAp/PLGA@IC (CHP@IC) scaffolds were prepared according to the CHP scaffold preparation process described above. Firstly, IC was synthesized according to our previous literature (Fig. S1) [35]. And different concentrations of IC were prepared in DMSO and then mixed with the DCM containing PLGA at a volume ratio of 3:10, and the homogeneous solution was used as the oil-phase. According to the above method, CHP@IC scaffolds were prepared. Scaffolds with different drug contents subjected to different polymerization times to balance the changes in performance. The obtained hydrogels were subjected to a sequential washing process with 20 % acetone and ultrapure water. Finally, the hydrogels were lyophilized to obtain CHP@IC scaffolds. CHP@IC_1.5,_ CHP@IC_3_ and CHP@IC_6_ were termed as 1.5 mg/mL, 3 mg/mL and 6 mg/mL concentration of IC in CHP@IC scaffolds. Furthermore, two control groups (CHP@ICA and CMA/HAp@IC) were prepared by *in situ* encapsulation of unmodified ICA in a CHP scaffold and directly encapsulation of IC in CM/HAp scaffold, respectively.

The mechanical property of the scaffolds was measured by dynamic mechanical analyzer (DMA, TA-Q800, USA). The swelling performance of scaffolds was measured by gravimetric change. The weight of the lyophilized scaffold was recorded as W_o_, then it was soaked in Tris-HCl buffer solution (pH = 7.4). After swelling balance, the scaffold was taken out and removed the residual solution on the surface. The wet weight of scaffold was recorded as W_w_, and the swelling ratio be calculated using the formula: Swelling ratio (%) = (W_w_ – W_o_)/W_o_ ∗ 100 %.

Meanwhile, the degradation behavior of scaffolds was measured by incubating in Tris-HCl containing 10 μg/mL collagenase I (Solarbio, China) at 37 °C. The weight (W) was recorded at preset time points. The rate of degradation was defined as (W_0_ − W)/W_0_ ∗ 100 %, where W_0_ is the initial wet weight of the sample.

The drug release behavior was detected by immersing scaffold into 1 mL Tris-HCl solution at 37 °C. At the point time, the solution was completely collected, and centrifuged. The supernatants were measured by ultraviolet spectrophotometer (UV–vis, Thermo Scientific, USA) at 360 nm. The encapsulation efficiency was determined through the following methodology: Microspheres were collected, washed, and subjected to centrifugation following the degradation of the scaffold. Thereafter, microspheres were dissolved in DMSO, and the absorbance was measured at 360 nm. Subsequently, the mass of the drug (W_2_) was calculated as a standard curve, and the encapsulation efficiency of the drug was calculated according to the following formula: W_2_/W_1_∗100 %, and W_1_ is the total amount of the drug added into the scaffold.

### 2.3 Cytocompatibility and osteogenic differentiation of scaffolds

After sterilization by ethylene oxide at 37 °C, scaffolds were incubated with 1 × 10^4^ cells/well BMSCs and HUVECs in 48-well plates using α-MEM (Corning, USA) and DMEM (Corning, USA) supplemented with 10 % FBS (Gibco, USA) and 1 % penicillin-streptomycin (Gibco, USA) for 1, 3 and 5 days with 5 % CO_2_ at 37 °C. At the point time, Cell counting kit (CA1210, Solarbio, China) was used to assess cell proliferation activity at 450 nm. Fluorescein diacetate (FDA, Sigma)/propidium iodide (PI, Sigma) and Phalloidin (CA1610, Solarbio, China)/DAPI (C0065, Solarbio, China) were used for staining Live (green)/dead (red) cells and cytoskeleton/cell nucleus, respectively. The images were photographed by confocal laser scanning microscope (CLSM, Zeiss, Germany). The spreading area of the cells was counted by image J software (NIH, USA).

To explore the effects of scaffolds on osteogenic differentiation, BMSCs were inoculated in scaffold at 48-well plates (1 × 10^4^ cells/well). On day 7 and day 14, the osteogenic differentiation potential of BMSCs was characterized by RT-qPCR (*Alp*, *Ocn*, *Runx2* and *Bmp2*) and ALP activity assay. On day 14, ALP staining and quantitative analysis as well as the IF staining of RUNX2 (ab236639, Abcam, UK) and OCN (ab93876, Abcam, UK) were performed.

### 2.4 Subcutaneous implantation of scaffolds

The animal experiment was approved by the animal ethics committee of Sichuan University (Approval No. KS2021698). Sprague-Dawley (SD) rats (males, 200–220 g) were used for subcutaneous implantation of scaffolds. After rats were anesthetized, CHP and CHP@IC group were randomly implanted on the left and right sides of the back of the rats to minimize individual differences in experimental animals. The scaffolds were collected at 3, 7, 14, and 21 days for histological analysis. All the samples were fixed with 4 % paraformaldehyde, Hematoxylin-eosin staining (H&E) was carried out by H&E staining kit (G1120, Solarbio, China). And Immunofluorescence staining (IF staining) of F4/80 (GB113373-100, Servicebio, China), iNOs (NB300-605, Novus, USA) and CD206 (ab300621, Abcam, UK) were performed to further verify the phenotype of the macrophages. The IF staining of CD31 (NB100-2284, Novus, USA) and α-SMA (GB111364-100, Servicebio, China) were performed to observe angiogenesis. The IF staining of ALP (ET1601-21, HUABIO, China) and OCN (ab93876, Abcam, UK) were conducted to evaluate osteogenesis of scaffold. The images were photographed by CLSM. The image J software was used for semi-quantitative statistics of all results.

### 2.5 Network pharmacology analysis

The Gene card database (https://www.genecards.org/), Swiss Target Prediction database (http://swisstargetprediction.ch/), Target net database (http://targetnet.scbdd.com/), and Pharm Mapper database (https://lilab-ecust.cn/pharmmapper/index.html) were employed to predict the potential targets of ICA [38,39]. The keywords “*osteogenesis*” and “*bone regeneration*” in the Gene card (https://www.genecards.org/) database were utilized to identify bone regeneration-related genes (Score >1). Subsequently, the potential targets of ICA were intersected with the aforementioned genes to ascertain the genes regulated by ICA in the process of bone regeneration. Following an analysis of the intersecting genes via the String database (https://string-db.org/), the Protein-Protein interaction network (PPI) was edited using the Cytoscape software. The degree value was then calculated by the Network Analyzer tool. In accordance with the aforementioned data, a GO enrichment analysis and a KEGG cluster enrichment analysis were conducted in the David database (https://david.ncifcrf.gov/) and the Metascape database (http://metascape.org/gp/index.html), respectively. Furthermore, the data were represented visually using the Wei Sheng Xin online tool (https://www.bioinformatics.com.cn/).

### 2.6 CHP@IC mediates immune response *in vitro*

RAW264.7 cells were seeded onto scaffolds in 48-well plates (5 × 10^3^ cells/well) and cultured with Dulbecco's Modified Eagle's Medium (DMEM, Corning, USA) for 5 days. The cell proliferation activity at 3 and 5 days was measured by CCK8. FDA/PI and Phalloidin/DAPI were used for staining cells and cytoskeleton, respectively. Then RAW 264.7 cells (5 × 10^4^ cells/well) were seeded on scaffolds and cultured with DMEM for 3 and 5 days. At each time point, the RT-qPCR was used to characterize the genes expression, including Arg*1*, *Inos*, *Il-10*, *Il-1β*, *Tnf-α*, *Cd206*. *Vegf*, *Tgf-β,* and *Bmp2*. Primer sequences of primers were listed in Table S1. Furthermore, the macrophage phenotype was characterized by flow cytometry (FC), cells were labeled with APC conjugated anti-mouse CD206 antibody (BioLegend, USA) and PE conjugated anti-mouse CD86 antibody (BioLegend, USA) and measured using a flow cytometer (BD Accuri C6, USA). On day 5, the expression of proteins was detected by western blotting (WB), IF staining and ELISA assay. For WB, cells were collected and lysed by RIPA with protease inhibitor (Solarbio, China). Protein (20 μg) from each specimen was loaded onto a 5 %–20 % polyacrylamide gel-plate and transferred to a PVDF membrane (Millipore Sigma) after electrophoresis. Then, the membrane was blocked with 5 % skimmed milk and incubated with iNOs (NB300-605, Novus, USA), ARG1 (16001-1-AP, Proteintech, UK), STAT3 (ET1607-38, HUABIO, China), phospho-STAT3 antibodies (S727) (ET1607-39, HUABIO, China) and GAPDH (ab8245, Abcam, UK) overnight. After incubation with HRP-conjugated secondary antibodies, visualization of the protein bands was performed by Bio-Rad ChemiDoc ECL detecting system (BioRad, USA) and analyzed by Image J software. The results were normalized to GAPDH expression. For IF staining of iNOs and CD206 were performed as previously described. The protein levels of VEGF and TGF-β in conditioned medium were examined with mouse VEGF and TGF-β ELISA kits (ExCellBio, Shanghai, China) according to the manufacturer's instructions.

The RAW264.7 cells were inoculated on the CHP@IC scaffold and treated with STAT3 inhibitor NSC74859 (SD4794, Beyotime, China) for 5 days, then the macrophage phenotype was characterized by FC. The expression of genes and proteins was assessed by WB and RT-qPCR assay.

### 2.7 The regulation of macrophages on angiogenesis of HUVECs

The medium from RAW 264.7 were harvested on day 5 and subsequently mixed with the DMEM complete medium at a ratio of 1:1 [40]. Additionally, the cell-free scaffold extract was collected and combined in a 1:1 ratio with complete medium. For cells migration, HUVECs (2 × 10^4^ cells/well) were seeded in the upper chambers and diluted conditioned medium was added in the lower chamber. After 24 h incubation, cells on the upper surface of the trans-well membrane were stained with 0.1 % crystal violet dye and photographed by microscope (LSM880, Germany). The number of migrated HUVECs was measured by Image J.

For angiogenic differentiation of HUVECs treated with conditioned medium, the expression of angiogenic differentiation-related genes and proteins was characterized by RT-qPCR and IF staining, respectively. In detail, HUVECs (5 × 10^3^ cells/well) were seeded in the 48-well plates with 0.6 mL diluted condition medium. After culture for 4 days, cells were collected and evaluated the angiogenic-related genes, including *Ang*, *Vegf*, *bFgf*, and *Fgfr1*. The sequences of primers used in this study are listed in Table S1. Then, the cells were fixed with 4 % paraformaldehyde to examine the relative expression level of VEGF (ET1604-28, HUABIO, China). Imaging was observed using CLSM and analyzed by image J. Meanwhile, for tube formation, HUVECs (3 × 10^4^ cells/well) resuspended in dilute conditioned mediums (100 μL) were inoculated on the surface of solidified Matrigel (50 μL, ABW Bio, China) in 96-well plates. After 8 h in the incubator, the cells were stained by FDA and images were captured by CLSM. Angiogenesis parameters (e.g., number of meshes, junctions, and segments, total segments length) in 3 randomly chosen fields were quantified using the Angiogenesis Analyzer macro in Image J software.

### 2.8 The regulation of macrophages on osteogenic differentiation of BMSCs

The medium from RAW264.7 and scaffold extract were harvested and subsequently mixed with the α-MEM complete medium at a ratio of 1:1, individually. For cells migration, BMSCs (2 × 10^4^ cells/well) were seeded in the upper chambers and the conditioned medium harvested on day 5 was added in the lower chamber. After 24 h incubation, the cells on the upper surface of the trans-well membrane were stained with 0.1 % crystal violet dye and photographed by microscope (LSM880, Germany). The number of migrated BMSCs measured by Image J software.

For osteogenic differentiation of BMSCs, the expression of osteogenic differentiation-related genes and proteins was characterized by RT-qPCR, ALP staining and quantitative analysis as well as IF staining, respectively. In detail, BMSCs (2 × 10^4^ cells/well) were seeded in the 48-well plates with 0.6 mL basic medium. After cell adhesion, the conditioned medium was used successively to replace the basic medium for further incubation. On day 4 and day 7, the osteogenic differentiation potential of BMSCs was characterized by RT-qPCR (*Alp*, *Ocn*, *Runx2* and *Bmp2*) and ALP activity assay. On day 7, ALP staining and quantitative analysis as well as the IF staining of RUNX2 (ab236639, Abcam, UK), BMP2 (ab284387, Abcam, UK), and OCN (ab93876, Abcam, UK) were performed. Moreover, in order to study the regulatory pathway of macrophage supernatant on osteogenic differentiation behavior of BMSCs, the relative protein levels of BMP2 (ab284387, Abcam, UK), OCN (ab93876, Abcam, UK), p-SMAD1/5 (9516, cell signaling technology, USA), β-Actin (ab6728, Abcam, UK), and GAPDH (ab8245, Abcam, UK) in BMSCs were measured via WB assay. Related genes (*Bmpr1*, *Smad 1*, *Smad 5* and *Smad 4*) were also characterized by RT-qPCR.

### 2.9 The treatment of CHP@IC in rat cranial bone defects

Sprague-Dawley (SD) rats (male, 200–220 g) were randomly divided into three groups: Blank group (defects only), CHP group, and CHP@IC group. After the experimental animals were anesthetized, the cranial epidermis and periosteum were bluntly separated using a scalpel, and a 5-mm full-thickness defect was created on both sides of the skull using a circular dental drill. Sterilized scaffolds were implanted into the defect site, and then the wound was closed. One week after the operation, the animals were sacrificed, and the materials at the defect site were collected for IF staining of iNOs and CD206 at the defect site. At postoperative 6 weeks, the rats were euthanized, the cranial was collected and fixed in 4 % paraformaldehyde. The defect was scanned topographically using the Quantum GX micro-CT imaging system (PerkinElmer, USA) with a scanning accuracy of 15 μm, and the data obtained were fully reconstructed and analyzed using Mimics software (Materialise, Belgium). H&E staining, IF staining, Immunohistochemistry (IHC) staining and Masson staining after decalcification were used to value the growth of blood vessels and bone repair. The image J software was used for semi-quantitative statistics of all results.

### 2.10 Statistical analysis

The data are presented as the mean ± SD. Statistical analysis was performed in GraphPad Prism 9.0 software using T-test, one-way ANOVA and two-way ANOVA. The mean of each column with the mean of every other column were compared. ∗: *p* < 0.05; ∗∗: *p* < 0.01; ∗∗∗: *p* < 0.001, and ∗∗∗∗: *p* < 0.0001.

## 3 Results and discussion

### 3.1 Fabrication and characterization of CHP and CHP@IC scaffolds

**Fig. 1:**
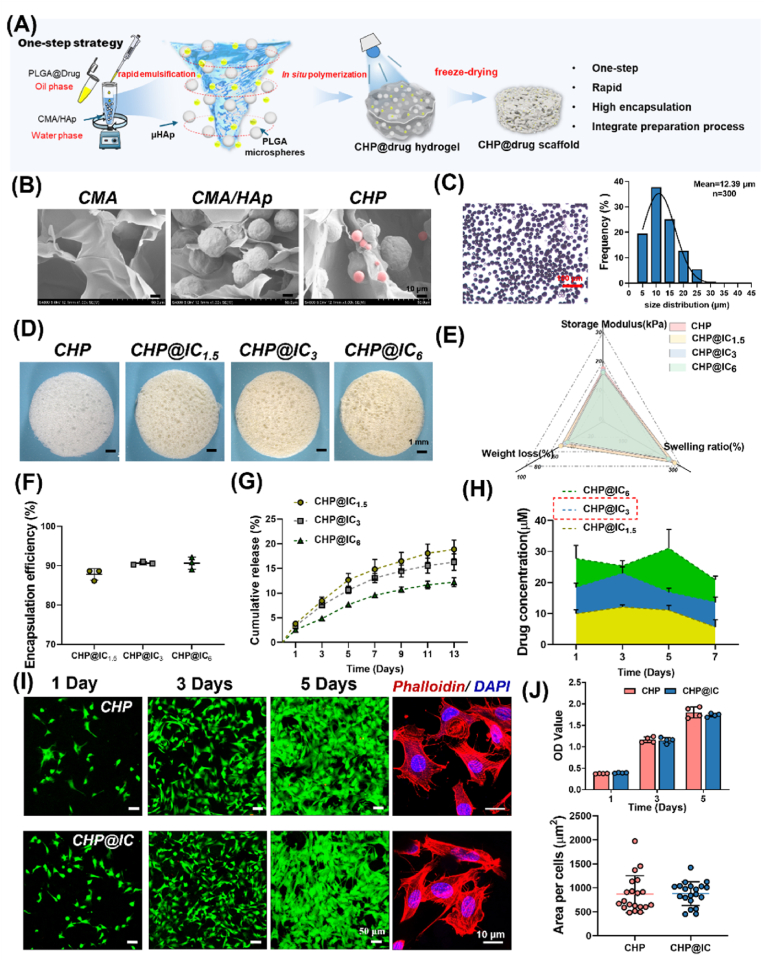
Preparation and characterization of CHP and CHP@IC scaffolds. (A) Design conception of the CHP@Drug scaffold. (B) Network structure of scaffolds and PLGA microsphere morphology (pink color) in biomimetic scaffolds from SEM images. (C) The gross view and particle size distribution of the *in situ* prepared PLGA microspheres (The transparent white balls are PLGA microspheres, and the opaque black balls are HAp in CHP). (D and E) The gross view and physicochemical properties of CHP@IC scaffolds. (F–H) The encapsulation efficiency, the cumulative release and the real-time concentrations of ICA in CHP@IC scaffolds. (I and J) The FDA/PI staining, phalloidin staining, CCK8 result and cell spread area of BMSCs cultured on the surface of CHP and CHP@IC scaffolds.

A new biomimetic scaffold containing *in situ* prepared PLGA microspheres was fabricated by a one-step strategy (Fig. 1A). As illustrated in Fig. 1B, SEM images demonstrated that CMA could be activated by blue light to rapidly form a photo-crosslinked network. HAp could be physically mixed and evenly distributed in the CMA network to form a biomimetic composite scaffold that closely resembling the composition of natural bone[34,41]. PLGA microspheres could be introduced into the CMA/HAp network via *in situ* emulsification, which offered a promising avenue for rapid drug encapsulation and delivery. Fig. 1C further presented the gross view and size distribution of the *in situ* prepared PLGA microspheres. The microsphere sizes were found to be normally distributed with an average size of approximately 12.39 μm and a high degree of convergence in the size distribution. Uniform microspheres can effectively regulate the drug release process [42]. Thus one-step strategy is expected to prepare porous biomimetic scaffolds and achieve rapid and efficient encapsulation and stable drug delivery.

ICA is an osteogenic active drug that is commonly utilized to enhance bone regeneration [27]. Local pharmacological intervention at the site of bone defects is expected to modulate physiological events during bone repair [16,43]. Given excellent compatibility of IC as well as the rapid drug loading and stable delivery process of CHP, three CHP@IC scaffolds with different IC loading amounts were prepared by a one-step strategy. As shown in Fig. 1D, the CHP@IC scaffolds exhibited a yellow color, and the color slightly deepened with the increase of drug content. Due to the volatilization of organic solvent and water during the preparation process, the surface of scaffolds showed a porous structure, which would be conducive to cell infiltration [44]. Moreover, due to the autofluorescence of ICA, it could be observed that the drug was uniformly distributed in the PLGA microspheres (Fig. S2). By adjusting the cross-linking time, the elastic modulus, swelling ratio and degradation process of three groups of CHP@IC scaffolds were close to each other (Fig. 1E and Fig. S3). And their drug encapsulation efficiencies were approximately 90 % (Fig. 1F). This was attributed to the *in situ* microsphere preparation process that avoided drug loss during curing and collection of the microspheres [45]. Additionally, all CHP@IC scaffolds exhibited controlled drug release processes compared with CMA/HAp@IC scaffold (Fig. 1G and Fig. S4A), indicating that the CHP@IC scaffolds not only avoided the sudden release of the drug, but also maintained the long-term drug release. Additionally, compared with CHP@ICA, CHP@IC had slower drug release and longer release cycle (Fig. S4B-4C). This was attributed to the formation of disulfide bonds among IC molecules. IC released from CHP@IC could only occur by breaking disulfide, amide or ester bonds [21,22,46].

A number of studies have demonstrated that the osteogenic activity of ICA is concentration-dependent, with optimal osteogenic activity occurring at a concentration of 10–20 μM [26]. The results of real-time concentrations of drug release from three groups of CHP@IC scaffolds at the first 7 days (Fig. 1H) demonstrated that CHP@IC_3_ was capable of maintaining safe and effective (10–20 μM) working concentrations. Therefore CHP@IC_3_ was selected as the drug-carrying scaffold in the subsequent experiment and all the subsequent references to CHP@IC represented CHP@IC_3_. The FDA/PI staining, the phalloidin staining and CCK8 results (Fig. 1I–J and Fig. S5) showed good cytocompatibility and cell spreading ability of CHP and CHP@IC scaffolds. Furthermore, Fig. S6 demonstrated that CHP@IC significantly promoted osteogenic differentiation of BMSCs by delivering an appropriate concentration of IC. In all, these results demonstrated that the one-step strategy could not only increase drug loading but also provide a stable drug release process. And CHP@IC scaffold had good cytocompatibility and osteogenic differentiation of BMSCs.

### 3.2 CHP@IC scaffold-induced biological responses

**Fig. 2:**
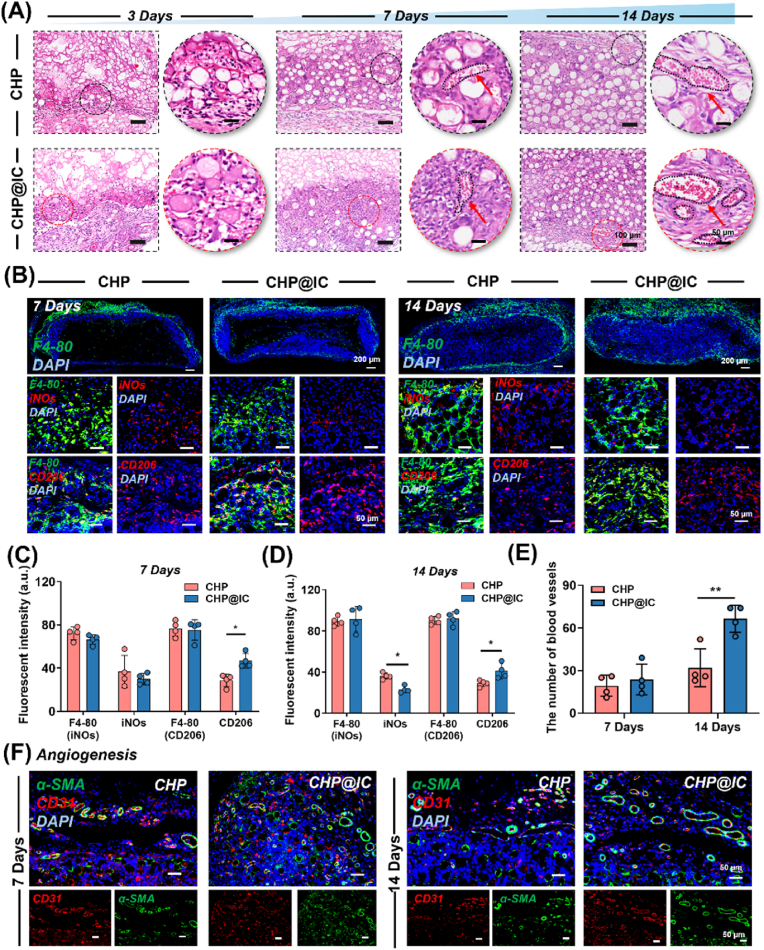
Scaffold-induced biological responses at 3, 7, 14 days. (A) The H&E staining for subcutaneous implantation of CHP and CHP@IC scaffolds at 3, 7, 14 days (red arrow: blood vessels). (B–D) The IF staining and quantitative analysis of F4-80, iNOs and CD206 at 7, 14 days (n = 4). (E) The number of blood vessels per scaffold (n = 4). (F) The IF staining of and CD31 and α-SMA at 7, 14 days (n = 4).

Subcutaneous implantation has become an important method to evaluate the biological events caused by scaffold implantation [47]. Accordingly, an evaluation of the subcutaneous biological responses induced by CHP and CHP@IC scaffolds in rats was conducted. As illustrated in Fig. 2A and Fig. S7A, H&E staining revealed that host cells had begun to accumulate around the scaffold and gradually penetrated into the interior after implantation for 3 days. In the magnified image, a variety of immune cells can be observed, including monocytes (bean-shaped nuclei), neutrophils (rod-shaped or lobular nuclei), and lymphocytes (oval nuclei), as deduced from histological features. By the 7 days, the interior of the scaffolds had been infiltrated with a substantial number of cells, and a small number of macrophages developing from monocytes could be observed. Furthermore, a substantial increase in vascular growth was evident at the site of cellular infiltration. These phenomena continued to develop until they were still observed after 14 days, and the number of vessels increased continuously with the depth of cellular infiltration in scaffolds. To the point is the fact that vessels in CHP@IC scaffold are becoming more numerous than that of CHP (Fig. 2E).

To directly identify macrophages and the influence of macrophage phenotypes after implanting scaffolds, the infiltration and phenotype of macrophages around the scaffolds were examined through IF staining for F4-80, iNOs, and CD206. As observed from Fig. 2B, F4-80 positive macrophages were noted around the scaffolds in both groups at 7 days post-implantation, and a certain percentage of macrophages subsequently spread to the interior of the scaffolds by 14 days. However, it was worth noting that macrophages around the two groups of scaffolds had different phenotypes, with relatively more iNOs^+^ macrophages around the CHP scaffolds and more CD206^+^ macrophages around the CHP@IC (Fig. 2B–D). In addition, due to the differences in blood vessels observed in H&E, the IF staining of CD31 and α-SMA was performed to further verify angiogenesis on day 7 and day 14. As shown in Fig. 2F, the number of new blood vessels in CHP@IC group was significantly higher than CHP group. The result of co-localized expression of CD31 and α-SMA confirmed that more mature blood vessels occupied the CHP@IC scaffold.

**Fig. 3:**
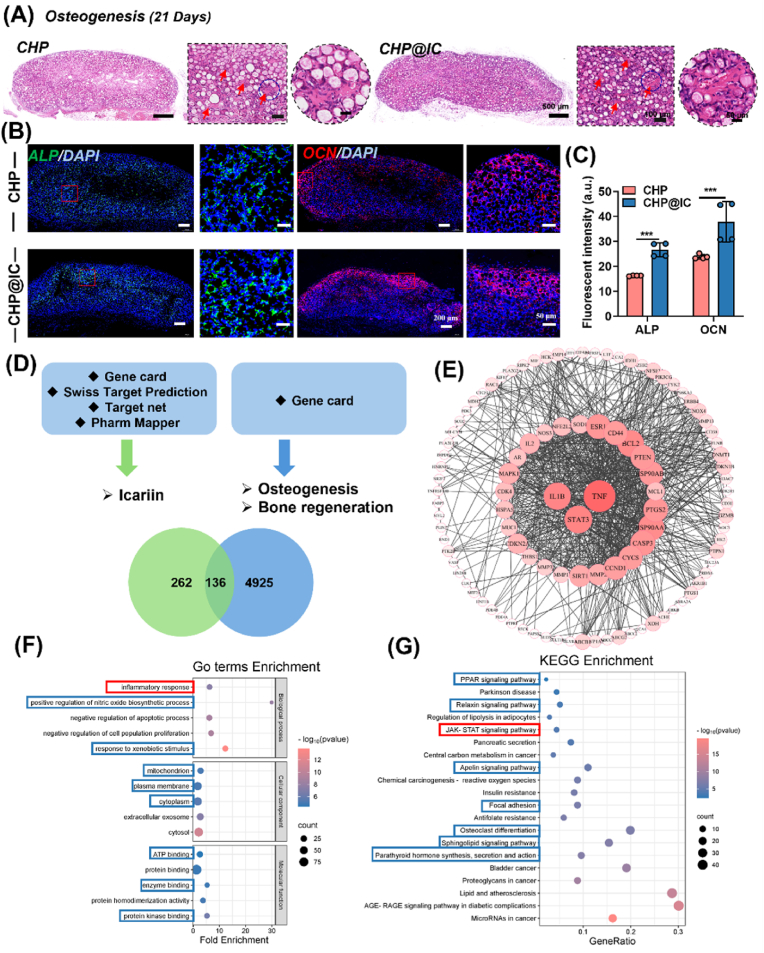
Scaffold-induced biological responses at 21 Days and network pharmacology analysis (A) The H&E staining for subcutaneous implantation of CHP and CHP@IC scaffolds at 21 days (Red arrow: bone-like matrix). (B) The IF staining and quantitative analysis of ALP and OCN in CHP and CHP@IC scaffolds at 21 days (n = 4). (D) The target of the ICA, the bone regeneration related gene and the intersecting genes of ICA and bone regeneration. (E) The PPI network of intersecting genes. (F) The GO enrichment analysis of intersecting genes. (G) The KEGG cluster enrichment analysis of intersecting genes.

When the implantation period of the scaffolds was extended to 21 days, the scaffolds were completely occupied by cells (Fig. 3A) and the process of cellular infiltration occurred at a faster rate in the CHP@IC group (Fig. S7B). The number of vessels within scaffolds began to decline on 21 days, accompanied by a substantial accumulation of matrix. In view of the excellent osteo-inductive activity of HAp and ICA, two kinds of osteogenic marker proteins ALP and OCN were stained by IF staining. As Fig. 3B and C denoted that ALP, a marker of early osteogenesis, was highly expressed in the interior of scaffolds. While OCN, a marker of late osteogenesis, was highly expressed at the edge of scaffolds. This was related to the sequence of cellular infiltration. Meanwhile, the two kinds of marker proteins possessed a higher expression in the CHP@IC scaffold than that of in the CHP scaffold.

Numerous studies have shown that the implanted biomaterials preferentially promoted the formation of M1-type macrophages to trigger local inflammation, drive phagocytic function, and secrete pro-inflammatory factors. Subsequently, the increase of M2 macrophages not only prevents the occurrence of excessive inflammation but also promotes tissue regeneration and repair in a variety of ways [40,44,48]. These results of subcutaneous implantation also show that CHP@IC scaffolds possess such potential to mobilize macrophage phenotypic shifts in a timely manner and also to regulate subsequent biological events such as angiogenesis and osteogenic differentiation, but the intrinsic link between these events remains to be further specified. A large number of literatures confirm that ICA directly promotes osteogenic differentiation, but it has also been suggested that this effect is linked to immune modulation [24,27,49]. Thus, some issues remain to be addressed. Firstly, it is necessary to identify the pathway that CHP@IC affects bone regeneration through immune regulation. Secondly, it is important to elucidate the internal relationship between the changed immunoinflammatory response and subsequent angiogenesis and osteogenesis in CHP@IC mediated the bone repair process.

### 3.3 Network pharmacology analysis

To predict the mechanism of action of CHP@IC in bone regeneration, we conducted an analysis of potential targets of ICA by network pharmacology [38,39,50]. A total of 136 intersecting genes (Table S2) were obtained by matching the keywords “*osteogenesis*” and “*bone regeneration*” with ICA targets in online web databases in Fig. 3D. PPI network was edited using the Cytoscape software, and the core targets were demonstrated to be TNF, IL-1β, and STAT3 based on degree value in Fig. 3E. TNF is a common inflammatory factor that affects RANKL-mediated osteoclastic activation [51]. Genes related to TNF in the intersecting genes include TNF, TNFSF11(RANKL), and TNFRSF11A, TNFRSF11B(OPG), which are genes related to ICA to activate/inhibit osteoclast activation. IL-1β is also a common inflammatory factor, and its activation is closely linked to the inflammatory response. In addition to this, ICA is able to modulate IL-1β-induced osteoarthritis [20]. Signal transducer and activator of transcription 3 (Stat3) is implicated in a variety of biological responses in organisms, including cell proliferation, differentiation and inflammatory responses [52,53]. ICA has been reported to promote alveolar bone regeneration by promoting downstream OCN transcription via STAT3 and STAT3 bound to the OCN promoter [54]. It is worth noting that the commonality of the core targets points to the body's immune-inflammatory response.

Next, GO enrichment analysis in Fig. 3F was also used to demonstrate the enrichment of intersecting genes. ICA may regulate osteogenesis through biological processes such as inflammatory response, positive regulation of nitric oxide biosynthetic processes and response to xenobiotic stimulus, while ICA-regulated bone regenerative processes may be related to cellular components such as mitochondrion, plasma membrane and cytoplasm, where molecular functions may involve ATP, protein, enzyme and protein kinase bonding as well as protein homodimerization activity. We compared the literature and found that ICA indeed coordinated osteogenic and osteoclastic differentiation of PEEK scaffold with ICA coating by reducing the inflammatory response [24]. And ICA regulates the synthesis of NO stimulating the osteogenic differentiation of rat BMSCs [18]. In addition, ICA performs physiological functions related to a wide range of proteins (BMP, OCN, OPG, etc.), enzymes (ALP, TRAP, etc.), protein kinases (ERK, JNK and p38 kinase), and the transduction of various signaling pathways [25,55,56]. KEGG cluster enrichment analysis in Fig. 3G was used to specify the signaling pathways involved in the regulation of osteogenesis by ICA. These include the parathyroid hormone synthesis, secretion and action, osteoclast differentiation, focal adhesion and relaxin signaling pathway associated with osteogenesis and osteoblast genesis, and the JAK-STAT signaling pathway associated with inflammation regulation. As well as the sphingolipid signaling pathway, regulation of lipolysis in adipocytes and PPAR signaling pathway, which regulate lipogenesis and osteogenesis. These results suggest that ICA possesses numerous potential mechanisms for regulating bone regeneration [57]. And from the core targets in the PPI network, GO enrichment analyses, KEGG cluster enrichment analyses and the observations in the subcutaneous implantation of CHP@IC, the inflammatory response is an integral part of these mechanisms. Thus, it is necessary to further investigate the association and mechanism of CHP@IC scaffold-mediated immunoinflammatory response with the observed vascular-bone regeneration process.

### 3.4 CHP@IC regulates the immune microenvironment through the STAT3 signaling pathway

**Fig. 4:**
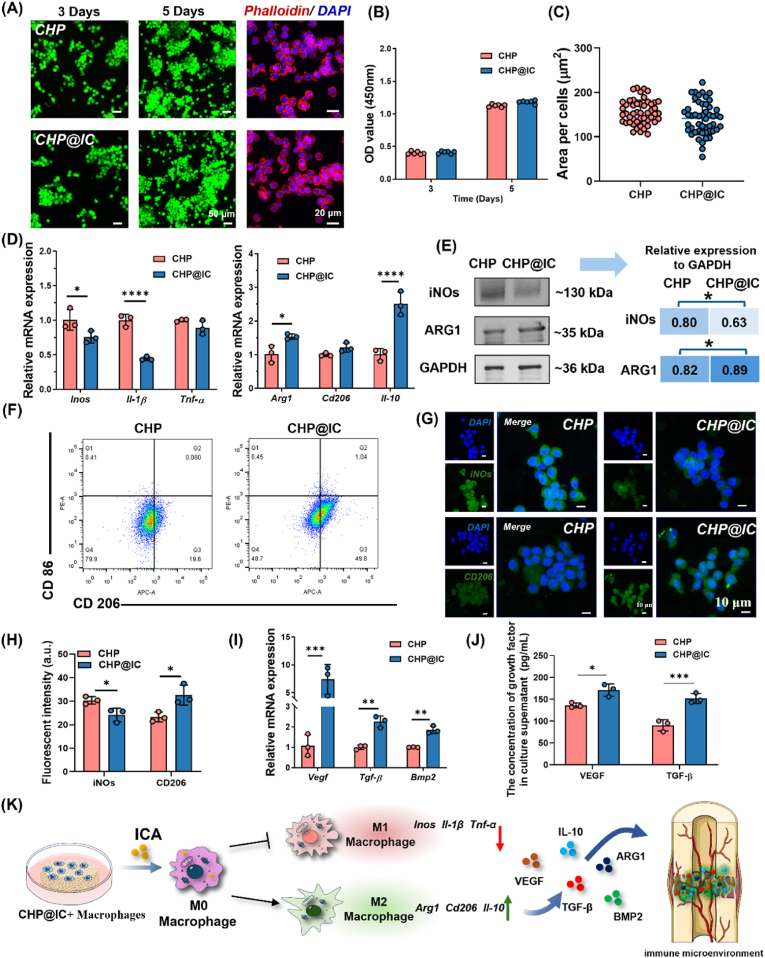
CHP@IC directs macrophage M2 polarization to regulate the immune microenvironment. (A–C) The FDA/PI staining, phalloidin staining, CCK8 result, and cell spread area of Raw264.7 cultured on the surface of CHP and CHP@IC scaffolds. (D) The RT-qPCR results of macrophage phenotypic transition at 5 days. (E)The WB results and the relative protein expression of iNOs, and ARG1 at 5 days. (F) The FC result of CD86 and CD206 at 5 days. (G) The IF staining of iNOs and CD206 at 5 days. (H) Quantitative analysis of iNOs, and CD206 fluorescent intensity (n = 3). (I) The RT-qPCR results of the cytokine in immune microenvironment at 5 days. (J) ELISA results of the cytokine at 5 days. (I) Schematic representation of the CHP@IC-mediated shift in the immune microenvironment.

In view of the results obtained in subcutaneous implantation as well as in the network pharmacology analysis, macrophages were used as the study object to comprehensively evaluate the immune-regulatory ability of CHP and CHP@IC scaffolds. Firstly, Raw264.7 was inoculated on the surface of the scaffold to observe its proliferation and spread process. The results (Fig. 4A–C) showed that macrophages could spread rapidly and exhibit a consistent proliferation process on both groups of scaffolds. Next, RT-qPCR results (Fig. S8A and Fig. 4D) revealed that the process of macrophage phenotypic transition, and there were no significant differences in genes associated with the phenotype in the early at two groups, while with increasing culture time, CHP@IC scaffolds significantly enhanced the expression of the M2-related genes (Arg*1*, *Il-10*, and *Cd206*) and decreased the expression of M1-related genes (*Inos* and *Il-1β*) in comparison with CHP groups on day 5 at the same total number of cells. Furthermore, compared with the CHP group, both and WB (Fig. 4E) and IF (Fig. 4G–H) results showed that CHP@IC group had significantly higher expression of M2 marker proteins (ARG1 and CD206) and lower M1 marker proteins (iNOs). In addition, the result of FC (Fig. 4F) showed that a significantly higher proportion of CD206-positive macrophages in the CHP@IC group than in the CHP group. These results demonstrated that the CHP@IC scaffold progressively modulated macrophage phenotypic shifts through ICA delivery.

With the rise of osteoimmunology, the regulation of osteogenic immune microenvironment by macrophages has received much attention [8,9]. Some studies have shown that macrophages change to M2 phenotype in response to the microenvironment, which not only down-regulates inflammation by secreting anti-inflammatory factors (IL-10, ARG1) to promote bone repair, but also activates the vascular-bone regeneration cascade through the release of growth factors (TGF-β, BMP2 and VEGF) [14,40]. This finding is consistent with our results indicating that macrophage type shifts impact subsequent vascular-bone regeneration processes in subcutaneous implantation. Hence, in order to further evaluate differences in the immune microenvironment constructed by scaffold-treated macrophages, RT-qPCR and ELISA were used to detect the intracellular/extracellular expression levels of typical growth factors. The RT-qPCR results (Fig. S8B and Fig. 4I) showed that compared with the CHP group, although only the expression of *Bmp2* in macrophages was up-regulated in CHP@IC group on day 3, CHP@IC group significantly up-regulated the expression of *Vegf*, *Tgf-β*, and *Bmp2* in macrophages on day 5. Similarly, on day 3, the ELISA results (Fig. S8C) showed that two groups had similar levels of VEGF and TGF-β secretion. However, on day 5, the VEGF and TGF-β secreted by macrophages in the CHP@IC group were significantly higher than those in the CHP group (Fig. 4J).

**Fig. 5:**
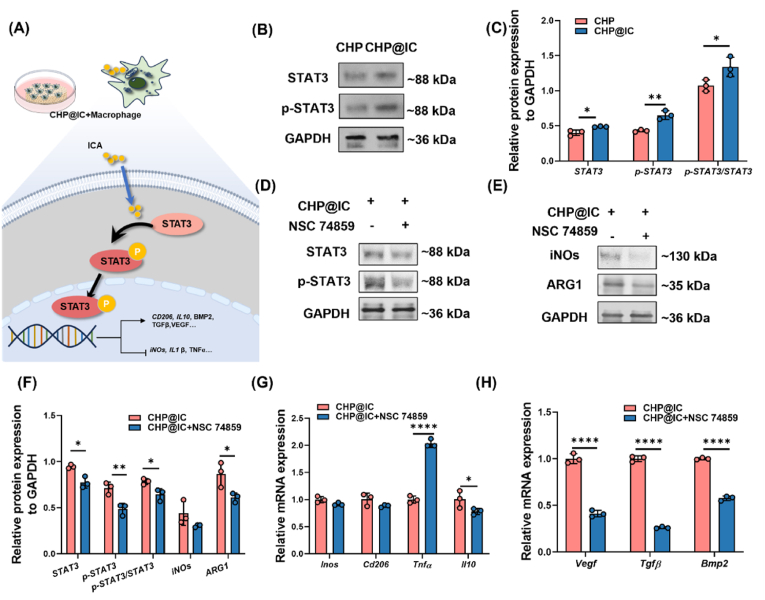
CHP@IC activates the STAT3 signaling pathway. (A) Schematic representation of CHP@IC activation of the STAT3 signaling pathway. (B–C) The WB results and the relative protein expression of STAT3, p-STAT3 and p-STAT3/STAT3 treated with CHP@IC scaffold. (D) The WB results of STAT3, p-STAT3 and p-STAT3/STAT3 treated with/without NSC 74859. (E) The WB results of iNOs and ARG1 treated with/without NSC 74859. (F)The quantitative analysis of protein expression with/without NSC 74859. (G–H) The RT-qPCR results of macrophages treated with/without NSC 74859.

STAT3 is a cytoplasmic transcription factor that plays a role in a variety of biological processes, including cell proliferation, differentiation, anti-apoptosis, inflammatory response, and angiogenesis [53]. Furthermore, research has demonstrated that the activation of the STAT3 pathway is associated with M2 polarization in macrophages [52]. The results of network pharmacology analysis showed that STAT3 is a potential target of ICA enrichment to a high degree, while the CHP@IC scaffold can modulate the immunoinflammatory response associated with macrophage phenotype *in vivo* and *in vitro*. Therefore, we firstly assessed the protein expression of STAT3 and phosphorylated STAT3 (p-STAT3) after CHP@IC treatment by WB assay and confirmed that reasonable ICA delivery in CHP@IC markedly upregulates the expression of STAT3, p-STAT3, and p-STAT3/STAT3 in RAW264.7 (Fig. 5B–C). Subsequently, to further validate the effect of STAT3 signaling pathway activation on macrophage M2 polarization as well as immune microenvironment alterations, a small molecule inhibitor for STAT3 (NSC 74859) was employed to inhibit the activation of the STAT3 signaling pathway in macrophages. The expression of genes and proteins related to polarization, as well as the expression of immune microenvironment-related genes, was then examined. When the STAT3 pathway was inhibited, STAT3, p-STAT3 and p-STAT/STAT3 were significantly down-regulated (Fig. 5D–F). FC was used to detect the macrophage phenotype after NSC 74859 treatment. The results in Fig. S9 showed that after the addition of the inhibitor, the number of M2-positive macrophages decreased significantly. Furthermore, alterations were observed in the expression of genes and proteins associated with polarization. The gene *Tnf-α*, which is linked to the M1 macrophage phenotype, demonstrated a notable increase in expression, while the genes *Il-10* and the protein ARG1, which are associated with the M2 macrophage phenotype, exhibited a decline in expression (Fig. 5E–G). Additionally, the corresponding immune microenvironment underwent a substantial alteration, with a marked decrease in the expression of *Vegf*, *Tgf-β*, and *Bmp2* (Fig. 5H). These provide compelling evidence that the STAT3 signaling pathway plays a pivotal role in the process of CHP@IC-mediated macrophage polarization and alteration of the immune microenvironment (Fig. 5A).

These findings demonstrate that the CHP@IC scaffold promoted the M2 polarization of macrophages via activation of the STAT3 signaling pathway (Fig. 5A), establishing a favorable immunomodulatory microenvironment that subsequently upregulated angiogenesis- and osteogenesis-related factors (Fig. 4K), which would influence the process of vascularization and bone regeneration.

### 3.5 CHP@IC mediated macrophages to improve the angiogenesis of HUVECs

**Fig. 6:**
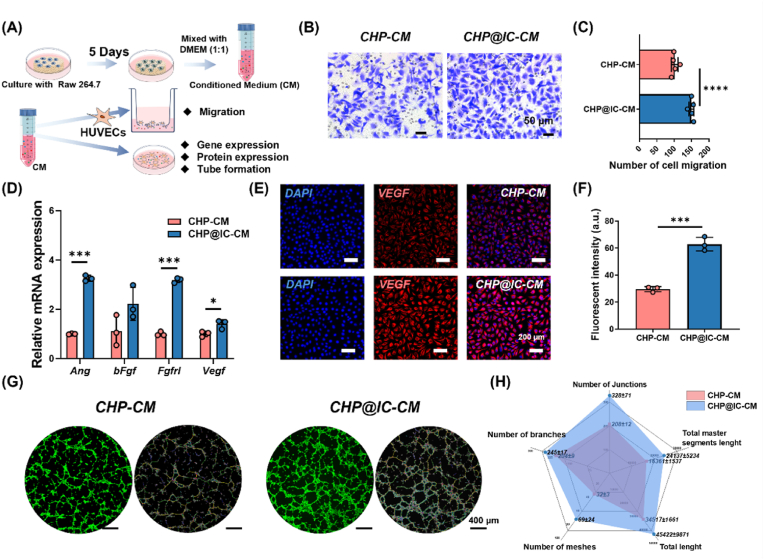
CHP@IC mediated macrophages to improve the angiogenesis of HUVECs. (A) The illustration of experimental design. (B–C) Trans-well assay images and the quantitative analysis of migrated HUVECs treated with CHP-CM and CHP@IC-CM (n = 5). (D) The RT-qPCR results of angiogenesis (n = 3). (E–F) The IF staining and quantitative analysis of VEGF treated with CHP-CM and CHP@IC-CM (n = 3). (G–H) The tube forming image of HUVECs and quantitative analysis(n = 3).

The above alteration of immune microenvironment *in vitro* suggested that CHP@IC might initiate immune-mediated angiogenesis via delivery of ICA. Angiogenesis is essential for the delivery of oxygen, nutrients, and metabolic wastes [58]. Studies also have shown that macrophage polarization triggered by biomaterials has the potential to regulate angiogenesis, thereby accelerating the regeneration and repair of bone tissue [59]. Toward this perspective, the effect of conditioned medium from macrophages treated with scaffolds (CHP-CM and CHP@IC-CM) at day 5 on the angiogenesis of HUVECs was studied (Fig. 6A).

Previous research had shown that following the concentration difference of growth factors (TGF-β and VEGF), endothelial cells changed their migration pathway [60]. The CHP@IC-CM induced more HUVECs migration than that in CHP-CM (Fig. 6B–C), which might be attributed to high expression of TGF-β and VEGF in CHP@IC-CM. Moreover, as shown in Fig. 6D–F, the CHP@IC-CM group had significantly higher expression of angiogenic differentiation-related genes (*Ang*, *bFgf, Fgfr1*, and *Vegf*) and VEGF protein than the CHP-CM group. Meanwhile, in contrast with the CHP-CM group, HUVECs organized into networks with significantly more capillary tube-like structures in the CHP@IC-CM group as Fig. 6G shown. Quantitatively, compared with CHP-CM groups, the CHP@IC-CM group significantly increased the tube formation parameters, such as the number of junctions, branches, and meshes as well as the total length and total master segment length in Fig. 6H. To more clearly demonstrate the function of the conditioned medium, cell-free material extracts were gathered and applied to culture HUVECs. The results (Figs. S10A–S10C) demonstrated that the extracts of CHP@IC scaffolds exhibited no notable impact on angiogenesis when compared to CHP scaffolds. While some studies have demonstrated that ICA can also stimulate angiogenesis, this effect is largely contingent on the concentration of the drug [61]. Whereas the effective concentration of the drug in the diluted extraction or conditioned medium is lower (Fig. S11), resulting in a diminished effect on angiogenesis. Therefore, our results indicated that the CHP@IC-treated macrophages secreted more pro-regenerative growth factors, which not only accelerated endothelial cell recruitment but also promoted angiogenesis.

### 3.6 CHP@IC mediated macrophages to promote osteogenic differentiation of BMSCs

**Fig. 7:**
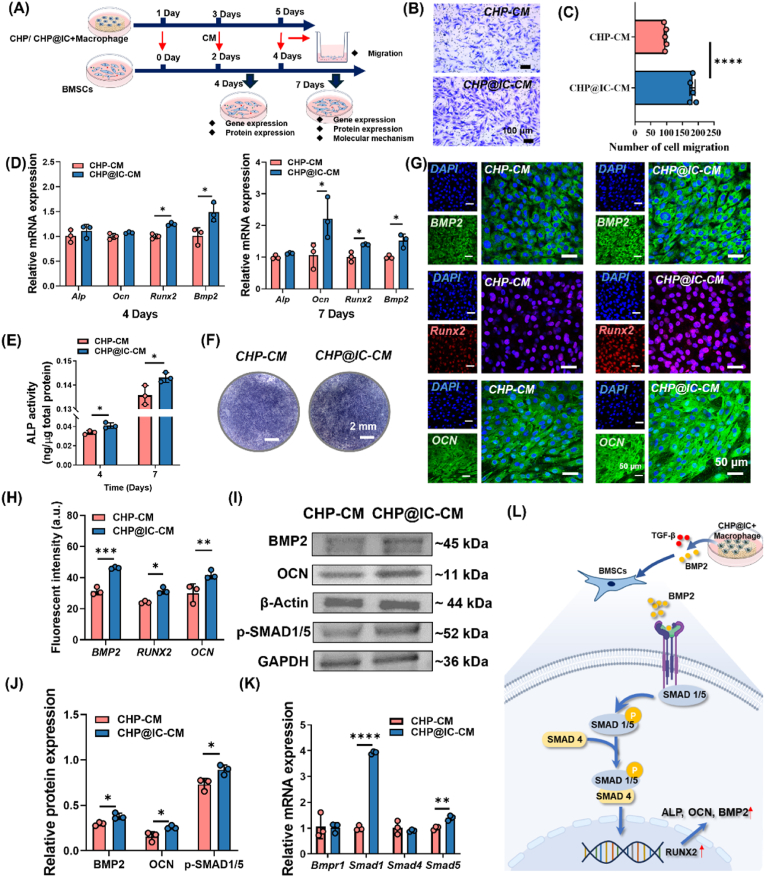
CHP@IC mediated macrophages to promote osteogenic differentiation of BMSCs (A) The illustration of experimental design. (B–C) Trans-well assay images and the quantitative analysis of migrated BMSCs treated with CHP-CM and CHP@IC-CM (n = 5). (D) The RT-qPCR results of osteogenic differentiation (n = 3). (E–F) The ALP staining and quantitative analysis treated with CHP-CM and CHP@IC-CM (n = 3). (G–H) The IF staining and quantitative analysis treated with CHP-CM and CHP@IC-CM (n = 3). (I–J) The WB results and the relative protein expression of BMP2, OCN and p-SAMD1/5 treated with CHP@IC scaffold (n = 3). (K) The RT-qPCR results about the mechanisms of osteogenic differentiation (n = 3). (L) Schematic representation about the mechanism of osteogenic differentiation treated with CHP@IC scaffold.

Increasing evidence shows that the crosstalk between macrophages and MSCs is indispensable for initiating new bone formation [40,59]. The above obtained results *in vivo* have shown that the macrophage-altered immune microenvironment may influence osteogenesis through the highly expressed pro-regenerative growth factors and cytokines. Thus, CHP-CM and CHP@IC-CM were also used to culture BMSCs to evaluate their modulatory effect on recruitment and osteogenic differentiation of BMSCs (Fig. 7A), respectively.

Previous research had shown that VEGF stimulated platelet-derived growth factor receptors (PDGFRs) to regulate MSC migration and proliferation [62]. And high expression of TGF-β and BMP2 also facilitated the migration of MSCs [40]. As shown in Fig. 7B, consistent with the migration results of HUVECs, the CHP@IC-CM group promoted the migration of BMSCs. This implied that the shift in macrophage phenotype altered the immune microenvironment and facilitated early stem cell recruitment, which was expected to promote new bone regeneration. Moreover, the RT-qPCR results showed that the expression of *Runx2* and *Bmp2* on day 4 and the expression levels of *Runx2*, *Bmp2*, and *Ocn* on day 7 in BMSCs treated with CHP@IC-CM were higher than those in CHP-CM treated (Fig. 7D). Meanwhile, there were also higher expressions of the early osteogenic marker ALP and osteogenic protein (OCN, BMP2 and RUNX2) in response to CHP@IC-CM stimulation (Fig. 7E–J). This might also be related to the presence of more BMP2 and TGF-β factors in the CHP@IC-CM. Similarly, the osteogenic capacity of the material extracts was evaluated. Overall, the material extracts of CHP@IC had some osteogenic activity (Fig. S12), but this ability was significantly weaker than that of the conditioned medium from macrophages. These findings illustrated that ICA orchestrated a precisely timed transition in the immunoinflammatory response, which subsequently exerts a profound influence on osteogenic differentiation.

Furthermore, the mechanism of promoting osteogenic differentiation of BMSCs by conditioned medium was further discussed. Considering the important role of the BMP2/Smad pathway in osteogenic differentiation [13,63], The RT-qPCR and WB were used to characterize the expression of proteins and genes related to the BMP2/Smad signaling pathway. As shown in Fig. 7D and 7I-7K, while the expressions of *Bmpr1* and *Smad4* remained unaltered, the expressions of *Bmp2*, *Smad1*, and *Smad5* exhibited a marked elevation at the 7 days, accompanied by a notable surge in BMP2 and phosphorylated Smad1/5 (p-SMAD 1/5) in protein levels. Therefore, these results suggested that CHP@IC-treated macrophages promoted osteogenic differentiation of BMSCs via BMP2/Smad1/5 pathway (Fig. 7L).

### 3.7 CHP@IC accelerated bone repair through activating vascular-bone regeneration cascade *in vivo*

**Fig. 8:**
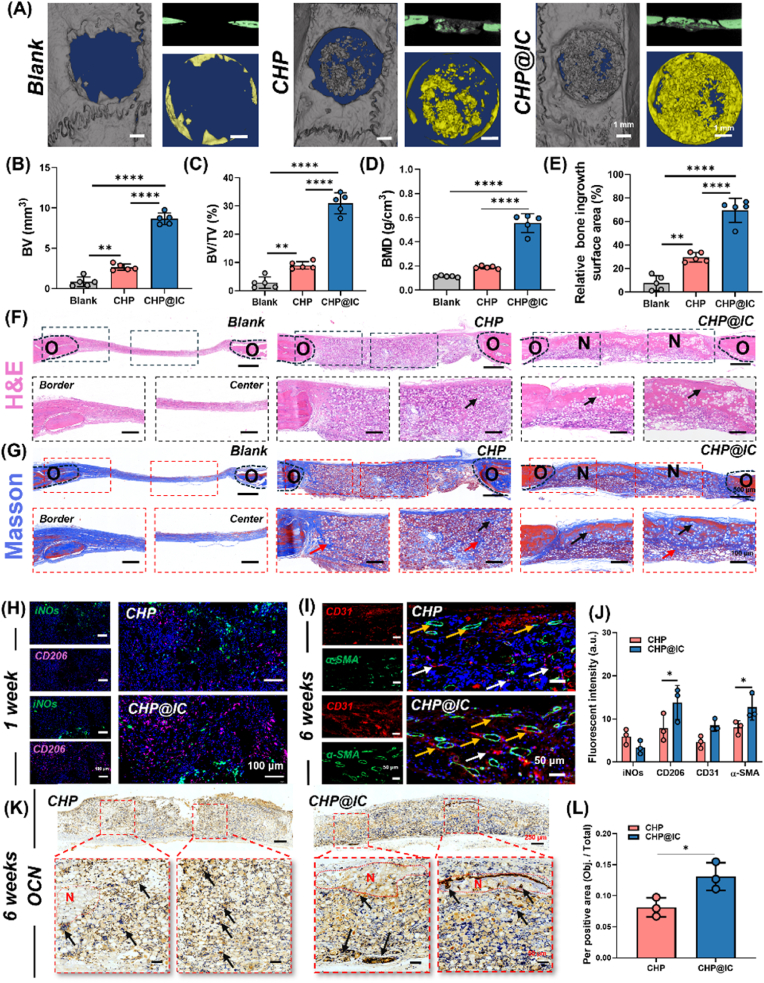
CHP@IC accelerated bone defect repair. (A) Micro-CT analysis of bone defects after 6 weeks. (B–E) Newly formed bone volume (BV), bone mineral density (BMD),bone volume fraction (BV/TV), and relative bone ingrowth surface area in the cranial defects after 6 weeks (n = 5). (F–G) H&E and Masson staining of the defect sites after 6 weeks. (O: old bone, N: new bone, black arrow: new bone, red arrow: immature bone matrix). (H) The IF staining of iNOs and CD206 in the defect sites after 1 week (n = 3) (I)The IF staining of CD31 and α-SMA in the defect sites after 6 weeks (white arrow: immature vessel, yellow arrow: mature vessel) (n = 3). (J) the quantitative analysis of IF staining. (K–L) The IHC staining and quantitative analysis of OCN in the defect sites after 6 weeks (black arrow: OCN positive area, N: new bone) (n = 3).

The subcutaneous implantation and *in vitro* cell-level validation fully demonstrated that CHP@IC regulated the immune microenvironment through the STAT3 signaling pathway, initiating the vascular-bone regeneration cascade. Thus, the bone repair effect of CHP@IC scaffold was further evaluated by a model of rat cranial bone defect. The micro-CT results presented in Fig. 8A revealed that the blank group exhibited minimal new bone formation, while the CHP group demonstrated a slight increase in new bone formation. In contrast, the CHP@IC group displayed a significantly new bone formation, and the newly formed bone volume (BV), bone volume fraction (BV/TV), bone mineral density (BMD) and relative bone ingrowth surface area were subjected to quantitative analysis. The quantitative results in Fig. 8B–E showed that the new bone volume and bone mineral density of CHP@IC reached about 8.66 mm^2^ and 0.6 g/cm^3^, corresponding to about 30 % of the bone volume fraction, and achieved nearly 71 % of the relative bone ingrowth surface area, which was an approximately 3-fold and 10-fold significant increase in comparison with the CHP and blank groups. Furthermore, H&E and Masson staining were used for more comprehensive histological analysis in Fig. 8F–G. In the blank group, the defect site was entirely occupied by fibrous tissues. Within these fibrous tissues, there was minimal observable angiogenesis, and no evidence of bone formation. After CHP treatment, the cells were completely infiltrated into the scaffold and some neovascularization could be observed. The infiltrating cells secreted a large amount of matrix, and Masson staining showed that only a small amount of new bone was present in this matrix, with a large amount of immature bone matrix remaining. Following treatment in the CHP@IC group, a substantial quantity of new bone tissue was observed at the edge and center of the defect site. Furthermore, the edges of these new bone tissues were stained red by Masson staining, indicating that they were undergoing a transformation to a denser and more mature state [44]. Similarly, the IHC staining of OCN reveal a greater proportion of OCN-positive regions in the CHP@IC scaffold and new bone in Fig. 8K–L, indicating that osteogenesis was significantly accelerated in the CHP@IC group.

IF staining was further used to observe various biological events after scaffold implantation. The staining of iNOs and CD206 was firstly performed to observe the immune inflammatory response at the defect site after scaffold implantation (Fig. 8H). Consistent with the results observed in the subcutaneous and *in vitro* cell-levels, a greater number of CD206-positive macrophages were identified in the CHP@IC scaffold (Fig. 8J), which demonstrated that CHP@IC scaffold promoted M2 polarization of macrophages *in vivo* and established an immune microenvironment conducive to bone repair. Furthermore, to visualize the process of vascular regeneration, the IF staining for CD31 and α-SMA was performed in the CHP and CHP@IC groups (Fig. 8I–J). The results of staining and fluorescence semi-quantification showed that there were more vessels positively stained with CD31 and α-SMA in CHP@IC group compared with CHP group, which indicated more mature blood vessels in CHP@IC group. Collectively, these findings demonstrate that the CHP@IC enhances bone regeneration through coordinated modulation of immune-angiogenic-osteogenic cascade. Mechanistically, the scaffold primarily induces M2 macrophage polarization to establish a favorable immune microenvironment, thereby stimulating downstream regenerative processes involving coupled angiogenesis and osteogenesis.

## 4 Conclusion

In conclusion, we propose a one-step strategy to rapidly fabricate a drug-microsphere encapsulated scaffold (CHP@IC) in a continuous and rapid process, enabling drug-loaded microspheres *in situ* preparation, and simultaneous encapsulation within the scaffold. The CHP@IC scaffold not only avoided the burst release of IC but also maintained the long-term and stable release of IC. Moreover, the CHP@IC-induced M2 polarization of macrophages via STAT3 signaling pathway triggers the sequential activation of inflammation, angiogenesis, and osteogenesis to enhance bone regeneration.

Additionally, motivated by the successful development of CHP@IC, it is anticipated that this methodology can be extended to a broader range of drug encapsulation and delivery processes in future research. This approach may even extend to the concurrent delivery of multiple types of drugs or bioactive factors, with the objective of addressing the multifaceted challenges posed by complex bone defect environments. Moreover, by replacing the water phase (e.g. methacrylate modified hyaluronic acid, chitosan or gelatin, etc.) and solid phase particles (e.g. bioactive glass, functional micro/nano particles, etc.) in the composite scaffold, it can be applied in the repair of other tissues, such as cartilage, skin, cardiovascular and neural tissues, etc. Overall, this one-step strategy provides a novel and universal solution for drug delivery in tissue repair.

## CRediT authorship contribution statement

**Fengxin Zhao:** Writing – original draft, Validation, Investigation. **Fuying Chen:** Investigation, Conceptualization. **Tao Song:** Methodology. **Luoqiang Tian:** Validation. **Hang Guo:** Validation. **Dongxiao Li:** Supervision. **Jirong Yang:** Writing – review & editing, Funding acquisition. **Kai Zhang:** Supervision, Methodology. **Yumei Xiao:** Writing – review & editing, Supervision, Funding acquisition, Conceptualization. **Xingdong Zhang:** Supervision.

## Ethics approval and consent to participate

The animal experiment was approved by the animal ethics committee of Sichuan University (Approval No. KS2021698).

## Declaration of competing interest

Kai Zhang is an editorial board member for Bioactive Materials and was not involved in the editorial review or the decision to publish this article. All authors declare that there are no competing interests.
