# Supplementary material for: One-step strategy for fabricating icariin-encapsulated biomimetic Scaffold: Orchestrating immune, angiogenic, and osteogenic cascade for enhanced bone regeneration
Source: Bioact Mater. 2025 Jun 10;52:271–86. doi: 10.1016/j.bioactmat.2025.06.001 (PMC12182316; doi:10.1016/j.bioactmat.2025.06.001)
Supplement: Multimedia component 1 [file mmc1.docx]

Supplementary materials

**One-Step Strategy for Fabricating Icariin-Encapsulated Biomimetic Scaffold: Orchestrating Immune, Angiogenic, and Osteogenic Cascade for Enhanced Bone Regeneration**


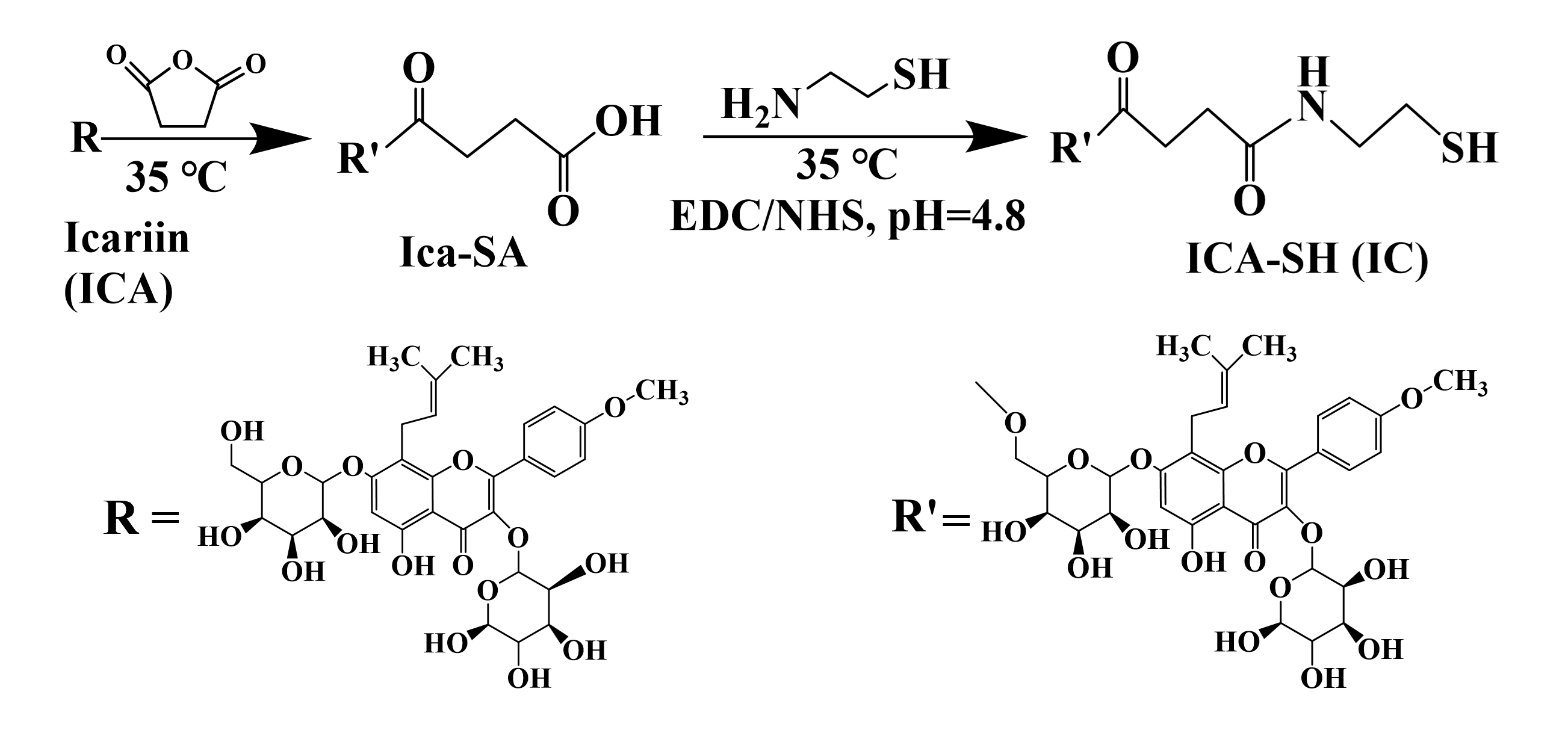


**Figure S1** Schematic representation of the preparation of ICA-SH (IC).


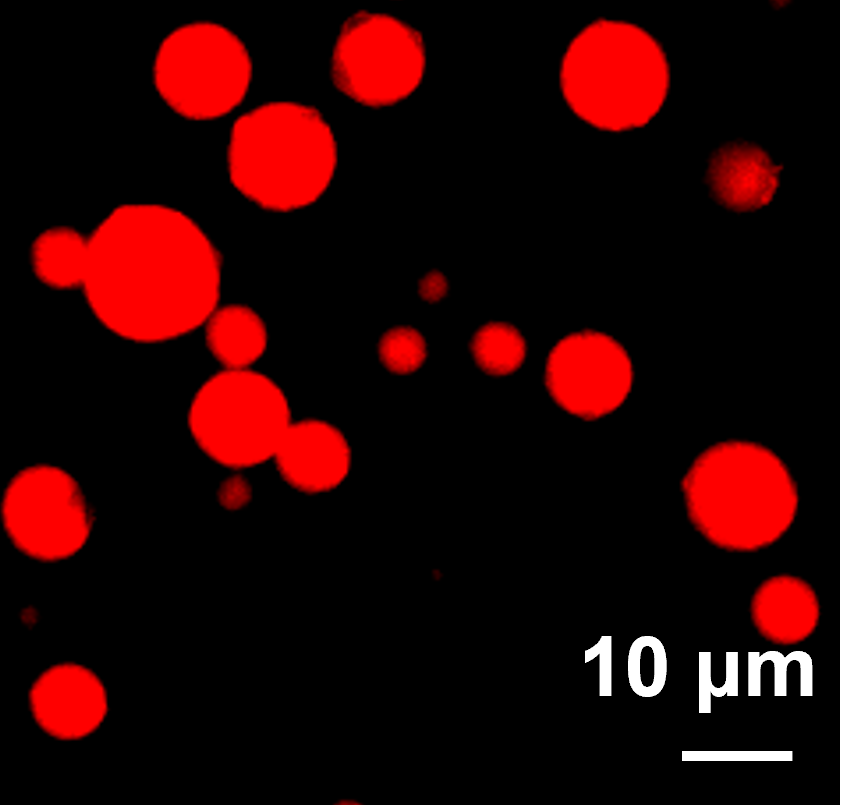


**Figure S2** The drug distribution in the PLGA microspheres.


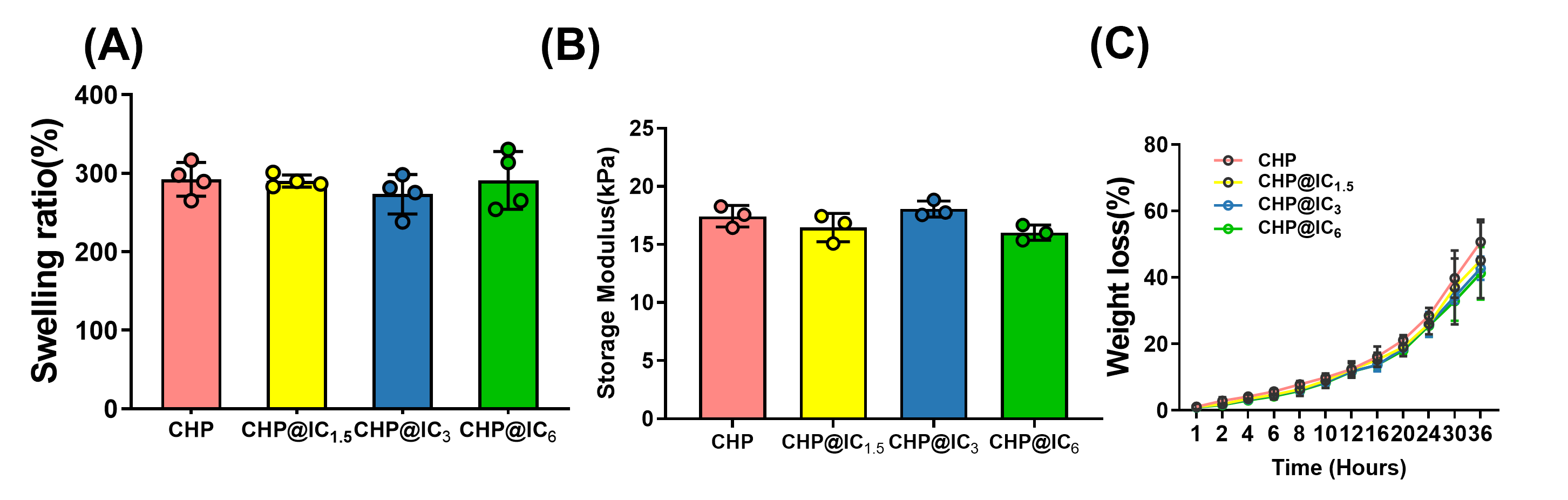


**Figure S3** The swelling ratio, the storage modulus and the the degradation behavior of CHP and CHP@IC scaffolds.


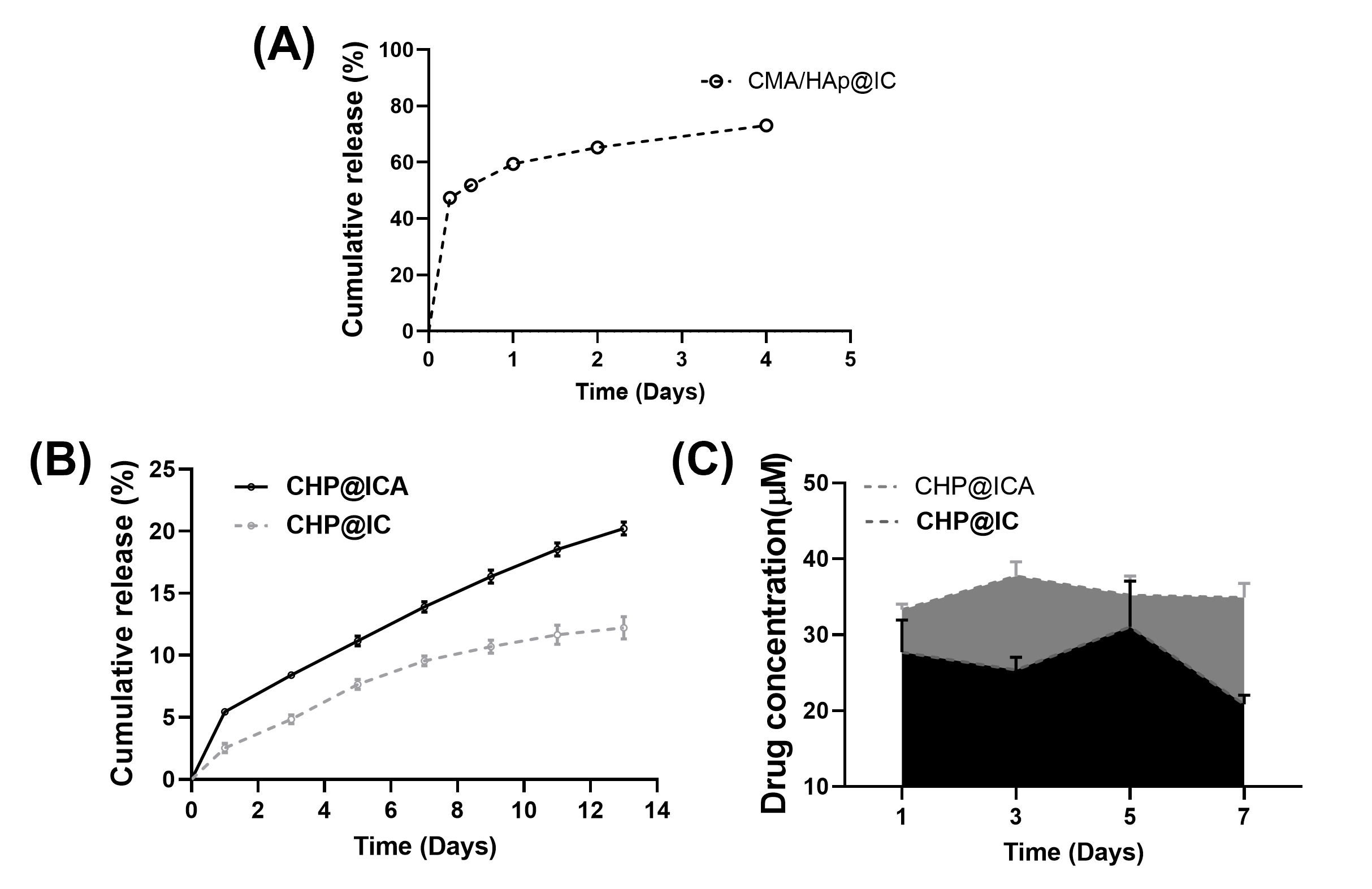


**Figure S4** (A) The cumulative release of CMA/HAp@IC. (B-C) The cumulative release and the real-time drug concentrations of CHP@ICA and CHP@IC scaffolds.


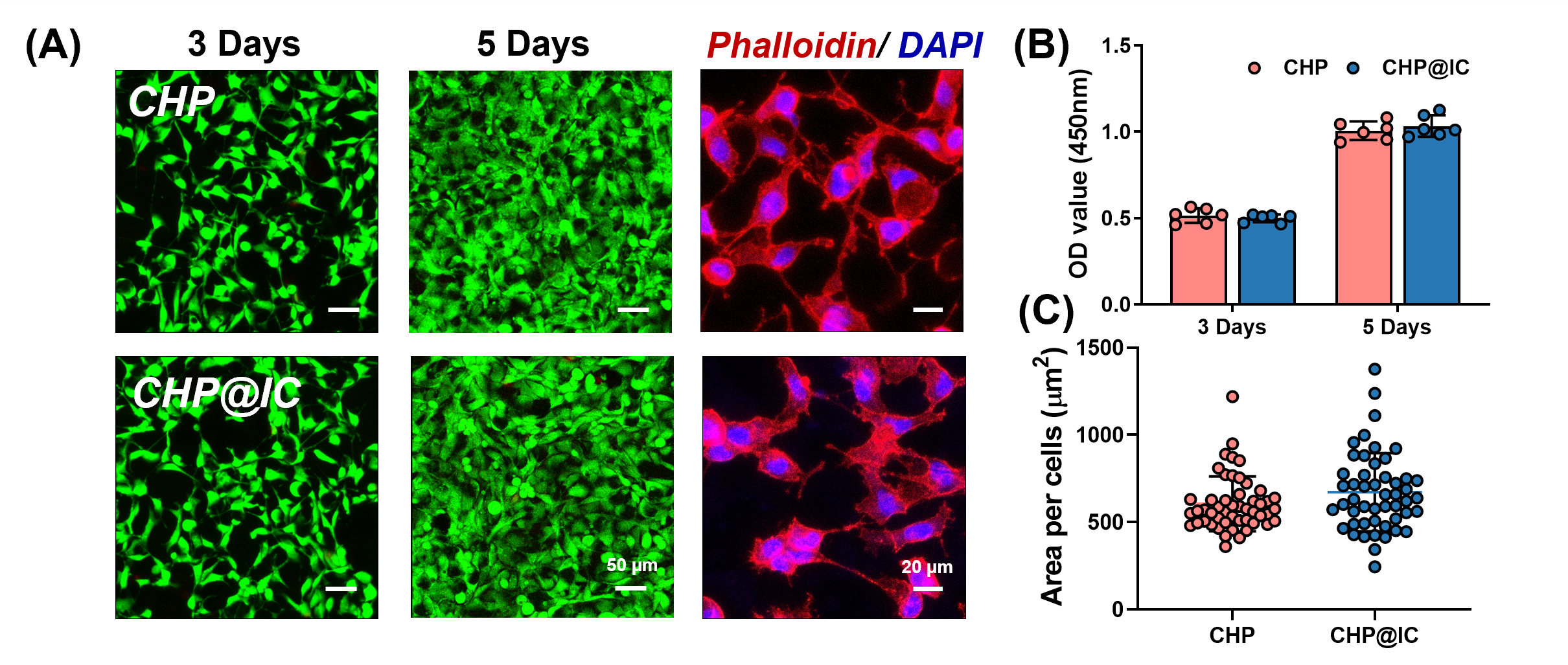


**Figure S5** (A)The FDA/PI staining, phalloidin staining, (B) CCK8 result and (C)cell spread area of HUVECs cultured on the surface of CHP and CHP@IC scaffolds.


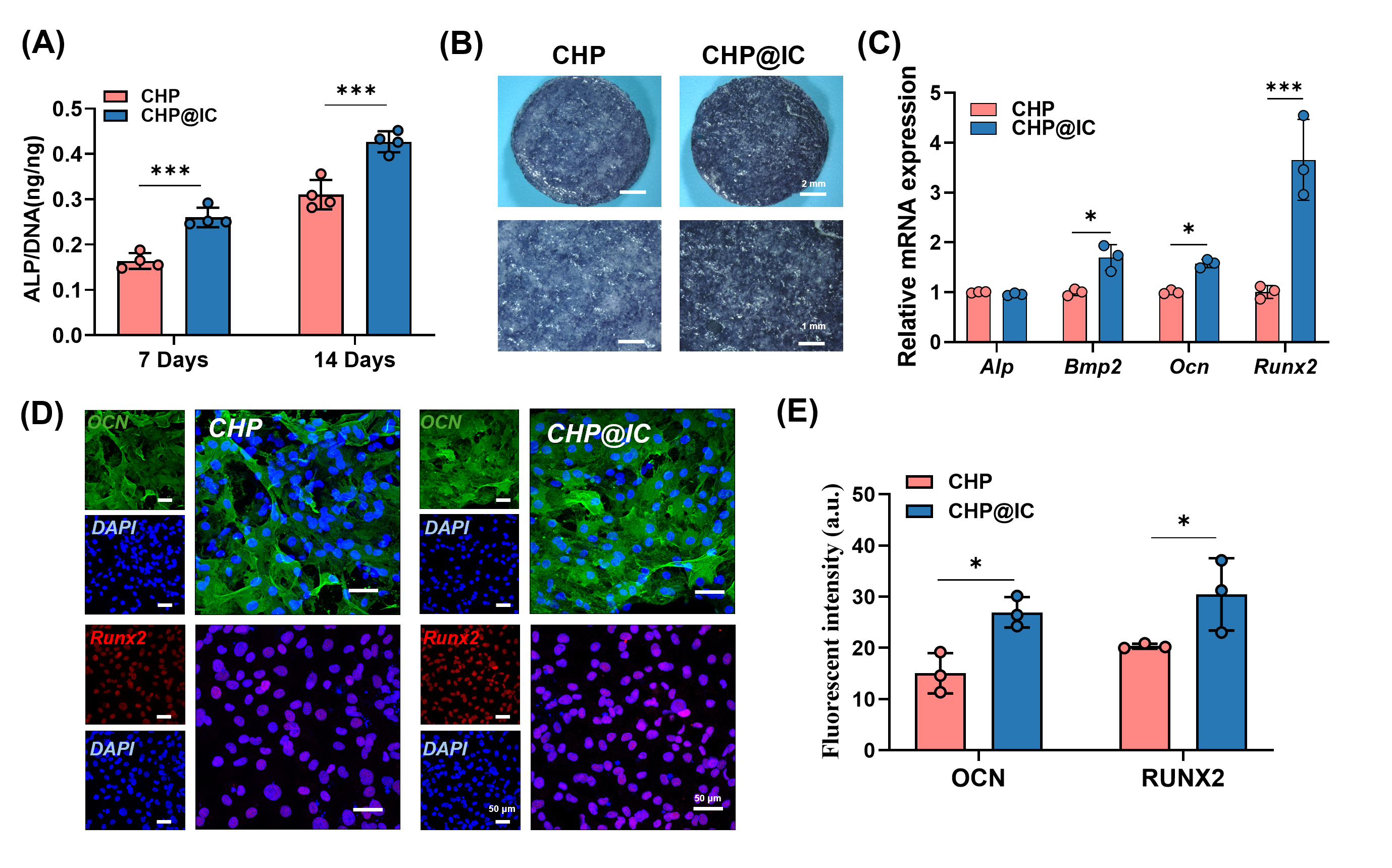


**Figure S6** the osteogenic differentiation of BMSCs cultured with scaffolds (A) The ALP quantitative analysis treated with CHP and CHP@IC at 7 days and 14 days (n=4). (B) The ALP staining at 14 days. (C) The qRT-PCR results of osteogenic differentiation 14 days (n=3). (D-E) The IF staining and quantitative analysis (n=3).


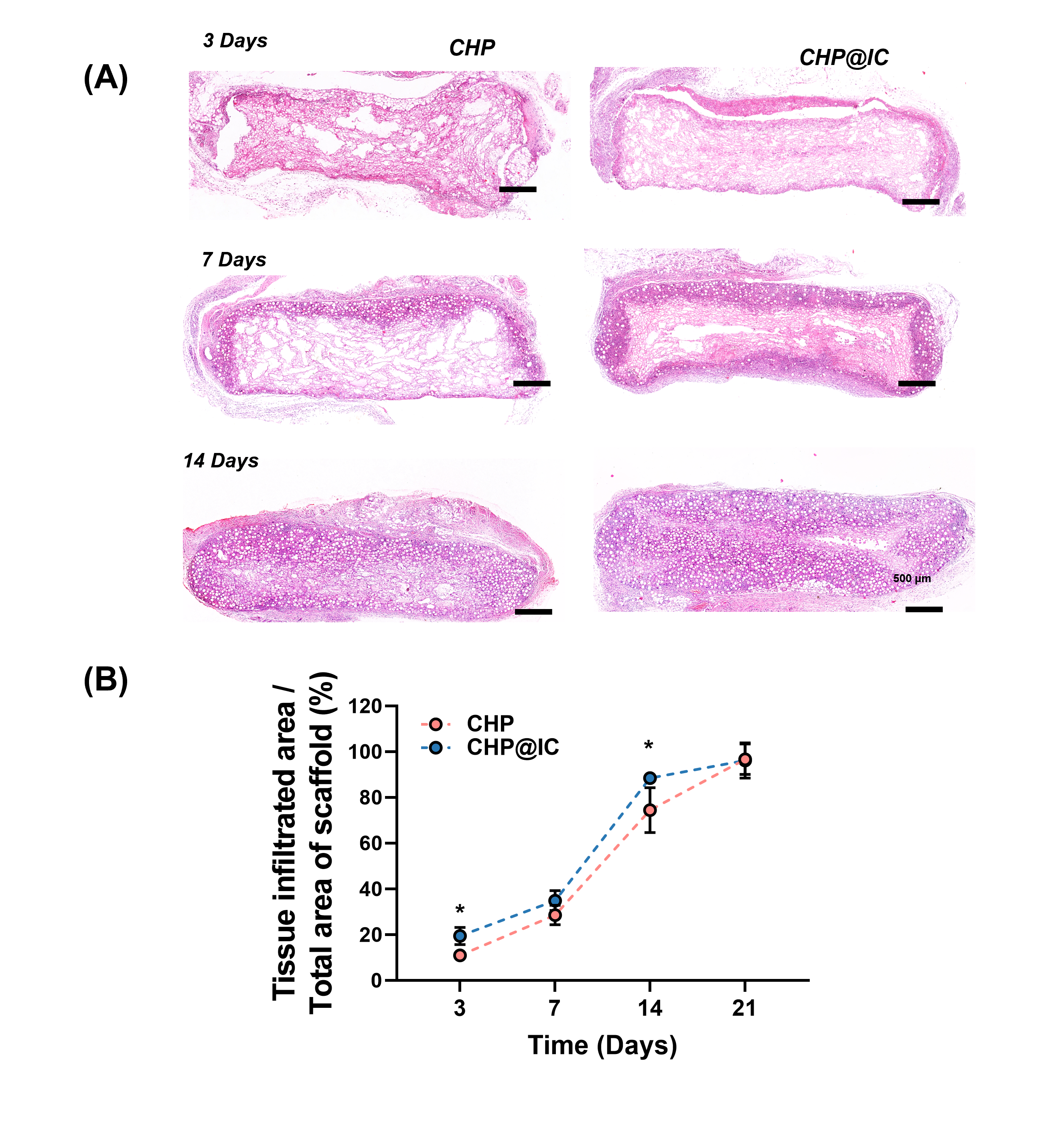


**Figure S7** (A) The goss view of H&E staining in subcutaneous implantation of CHP and CHP@IC scaffolds at 3, 7 and14 days (B) Relative area of tissue infiltration per scaffold (n = 4).


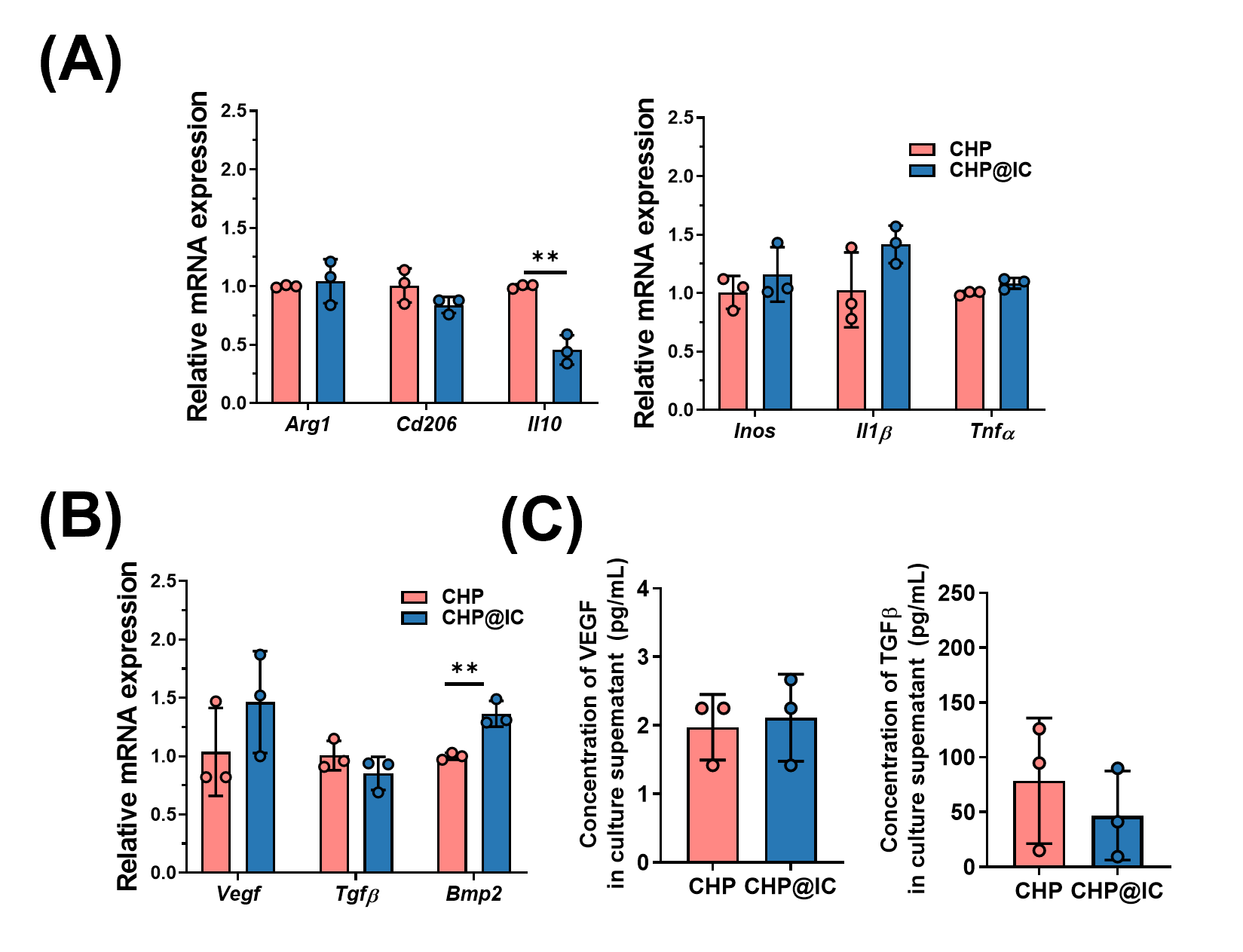


**Figure S8** (A) The qRT-PCR results of macrophage polarization at 3 days. (B) The qRT-PCR results of the cytokines in immune microenvironment at 3 days. (C) The Elisa results of the cytokines at 3 days (n=3).


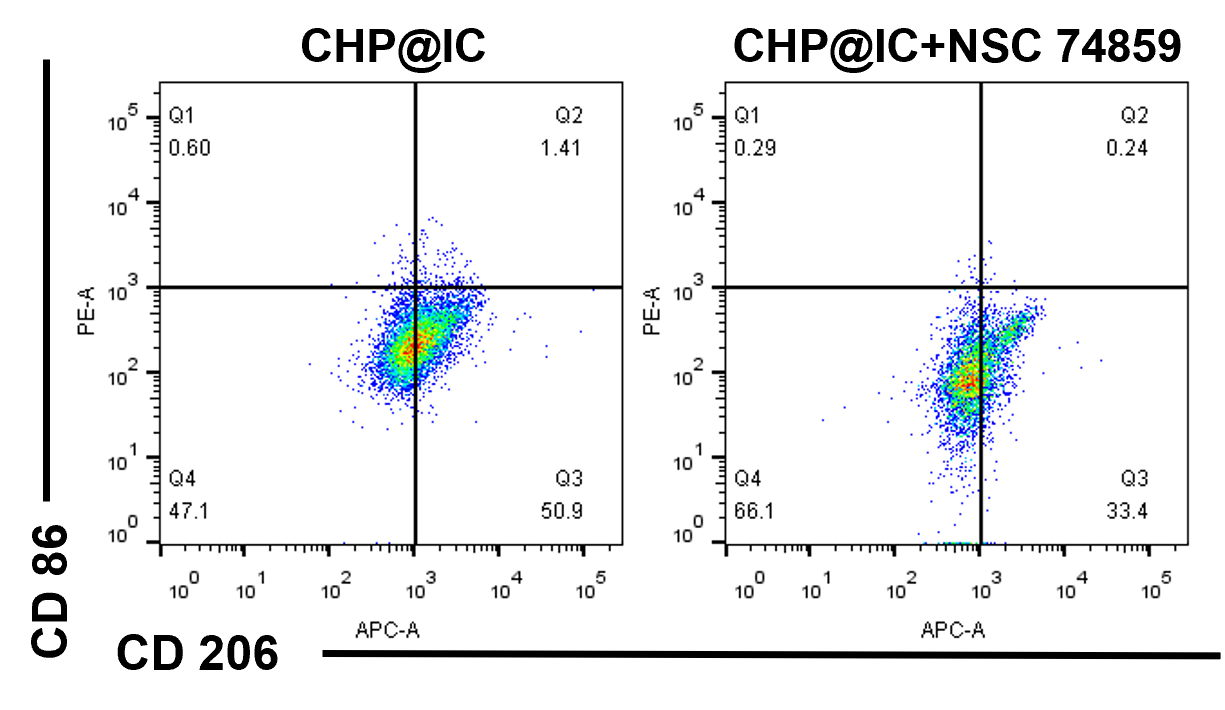


**Figure S9** (A) The FC results of macrophage phenotype with/without NSC 74859.


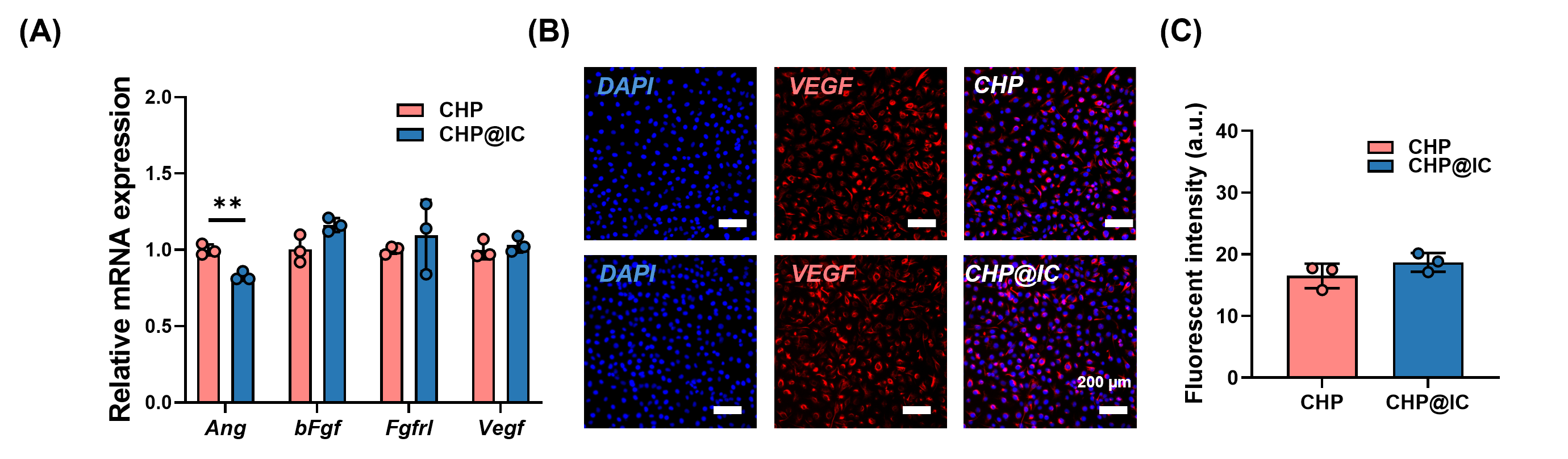


**Figure S10** The qRT-PCR results (A), the IF staining of VEGF (B) and the quantitative analysis of VEGF (C) of HUVECs treated with the extracts of CHP and CHP@IC scaffold at 4 days (n=3).


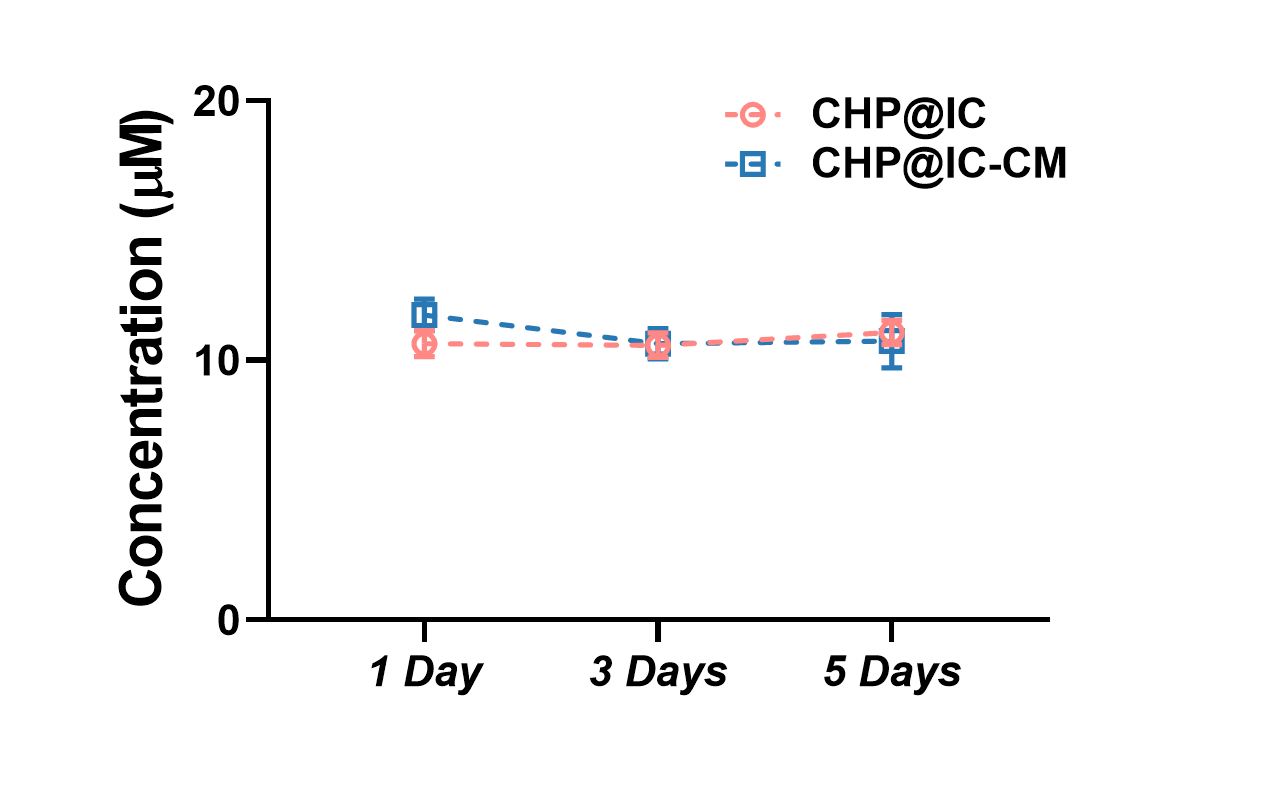


**Figure S11** IC concentrations in macrophage conditioned medium and material extract.


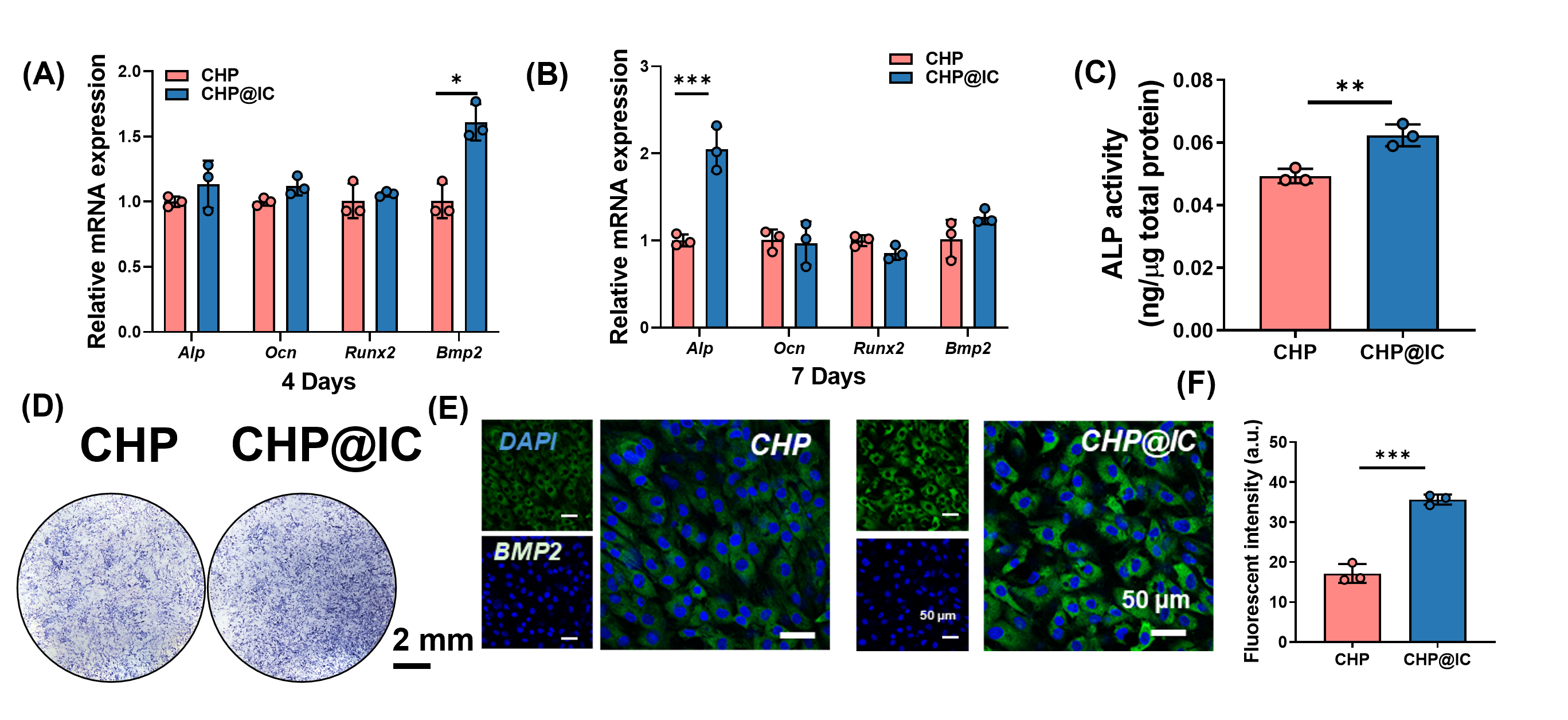


**Figure S12** (A-B)The qRT-PCR results of osteogenic differentiation of BMSCs treated with the extracts of CHP and CHP@IC scaffold at 4 and 7 Days (n=3). (C-D) The ALP staining and quantitative analysis of BMSCs treated with the extracts of CHP and CHP@IC scaffold at 7 Days (n=3). (E-F)The IF staining and quantitative analysis of BMP2 of BMSCs treated with the extracts of CHP and CHP@IC scaffold at 7 Days (n=3).

Table S1. The primer sequences of genes.

| *Gene (Mouse)* | Forward primer sequences (5’-3’) | Reverse primer sequences (5’-3’) |
| --- | --- | --- |
| *Tnfɑ* | TGGGAGTAGACAAGGTACAACCC | CATCTTCTCAAAATTCGAGTGACAA |
| *Inos* | CATTGGAAGTGAAGCGTTTCG | CAGCTGGGCTGTACAAACCTT |
| *Il1β* | AACCTGCTGGTGTGTGACGTTC | CAGCACGAGGCTTTTTTGTTGT |
| *Cd206* | GCACTGGGTTGCATTGGTTT | TGCAGGGTTGACATGAGACC |
| *Arg1* | GAACACGGCAGTGGCTTTAAC | TGCTTAGCTCTGTCTGCTTTGC |
| *Il10* | CACCTGCTCCACTGCCTTGCT | GGTTGCCAAGCCTTATCGGA |
| *Bmp2* | GGGACCCGCTGTCTTCTAGT | TCAACTCAAATTCGCTGAGGAC |
| *Vegf* | AGAGCAACATCACCATGCAG | CAGTGAACGCTCCAGGATTT |
| *Tgfβ* | GCAACATGTGGAACTCTACCAGA | GACGTCAAAAGACAGCCACTCA |
| *Alp* | CCAGCAGGTTTCTCTCTTGG | GGGATGGAGGAGAGAAGGTC |
| *Ocn* | AGCAGCTTGGCCCAGACCTA | TAGCGCCGGAGTCTGTTCACTAC |
| *Runx2* | GACTGTGGTTACCGTCATGGC | ACTTGGTTTTTCATAACAGCGGA |
| *Bmpr1* | CCTGTTGTTATAGGTCCGTTCTT | AGCTGGAGAAGATGATCATAGCA |
| *Smad1* | GGATGAGCTTCGTGAAGGGTTGG | GCAAGAGACGGAAGCCACAGG |
| *Smad4* | GTGACTGTGGATGGCTATGTGG | GCAACCTCGCTCTCTCAATCG |
| *Smad5* | ATCCCACCACTGTCTGTAAG | TTTATCCAGCCACTGAAGAGG |
| *Gapdh* | ACCCAGAAGACTGTGGATGG | CACATTGGGGGTAGGAACAC |

| *Gene (human)* | Forward primer sequences (5’-3’) | Reverse primer sequences (5’-3’) |
| --- | --- | --- |
| *Gapdh* | GAAGGTGAAGGTCGGAGT | GAAGATGGTGATGGGATTTC |
| *Vegf* | TACCTCCACCATGCCAAGTG | ATGATTCTGCCCTCCTCCTTC |
| *bFgf* | CAATTCCCATGTGCTGTGAC | ACCTTGACCTCTCAGCCTCA |
| *Fgfr1*  *Ang* | CCCGTAGCTCCATATTGGACA  CCAGGCCCGTTGTTCTTGAT | TTTGCCATTTTTCAACCAGCG  GGAAGGGAGACTTGCTCATTC |

Table S2. The intersecting genes of network pharmacology analysis.

|  | Gene name |
| --- | --- |
| *Intersecting genes* | CA4, AKR1B1, NOX4, CA2, PTGS2, RPS6KA3, ACHE, NMUR2, ADRA2A, CD38, TNF, IL2, ADORA1, PTGS1, SLC5A1, ABCG2, DNMT1, CYP1A2, PTPN1, ABCB1, MIF, CLK1, NR2F2, PLA2G1B, RIPK2, HSP90AA1, TYK2, IMPDH2, PLIN1, XDH, MCL1, GPR35, BLVRA, SULT1B1, EXOSC9, MEF2A, SCO2, MID1, CHKB, EIF4A1, CYCS, MMP2, SUOX, COG2, HK2, AOC3, GZMB, MYO6, RND1, AR, PRDX6, EPB41L3, ZEB2, MUC1, HNF1B, PLA2G2A, LTF, FLNB, CDK5R1, HCK, IDH1, MT-CYB, PTPRF, THBS1, ERBB4, SEC23A, CD3E, ESR1, PDK3, FABP3, HSP90AB1, PAPSS2, KIF11, CCND1, HDAC7, MDH2, BDNF-AS, PTK2B, NOS3, SOD2-OT1, H19, BCL2, PIK3CG, IL1B, HSPA5, LINC01672, CASP3, TMX2-CTNND1, NFE2L2, EMSLR, RAB4B-EGLN2, RNU6-1, CYTOR, TRA-TGC7-1, PTEN, RECK, MIR21, MAPK1, MMP1, MMP13, MMP3, MYL2, MIR216A, MIR133A2, MIR17, HCCAT5, MIR1469, MMP14, TNFSF11, TNFRSF11A, TNFRSF11B, PDE4A, PDE4B, SIRT1, SOD1, RAC1, VASP, XIST, MIR34C, SMAD5-AS1, SNHG1, ZFAS1, DNM3OS, TRP-AGG2-5, TRP-AGG2-6, CDK4, STAT3, CDKN2A, ABCC1, ABCC2, CDKN1B, RNF217-AS1, CD44, TFF1, RNY5, LINC02605 |
